# Homo- and Heteroassociations Drive Activation of ErbB3

**DOI:** 10.1016/j.bpj.2019.10.001

**Published:** 2019-10-09

**Authors:** Tímea Váradi, Magdalena Schneider, Eva Sevcsik, Dominik Kiesenhofer, Florian Baumgart, Gyula Batta, Tamás Kovács, René Platzer, Johannes B. Huppa, János Szöllősi, Gerhard J. Schütz, Mario Brameshuber, Peter Nagy

**Affiliations:** 1 Institute of Applied Physics, TU Wien, Vienna, Austria; 2 Department of Biophysics and Cell Biology, Faculty of Medicine, University of Debrecen, Debrecen, Hungary; 3 Department of Genetics and Applied Microbiology, Faculty of Science of Technology, University of Debrecen, Debrecen, Hungary; 4 Institute for Hygiene and Applied Immunology, Center for Pathophysiology, Infectiology and Immunology, Medical University of Vienna, Vienna, Austria; 5 MTA-DE Cell Biology and Signaling Research Group, Faculty of Medicine, University of Debrecen, Debrecen, Hungary

## Abstract

Dimerization or the formation of higher-order oligomers is required for the activation of ErbB receptor tyrosine kinases. The heregulin (HRG) receptor, ErbB3, must heterodimerize with other members of the family, preferentially ErbB2, to form a functional signal transducing complex. Here, we applied single molecule imaging capable of detecting long-lived and mobile associations to measure their stoichiometry and mobility and analyzed data from experiments globally, taking the different lateral mobility of monomeric and dimeric molecular species into account. Although ErbB3 was largely monomeric in the absence of stimulation and ErbB2 co-expression, a small fraction was present as constitutive homodimers exhibiting a ∼40% lower mobility than monomers. HRG stimulation increased the homodimeric fraction of ErbB3 significantly and reduced the mobility of homodimers fourfold compared to constitutive homodimers. Expression of ErbB2 elevated the homodimeric fraction of ErbB3 even in unstimulated cells and induced a ∼2-fold reduction in the lateral mobility of ErbB3 homodimers. The mobility of ErbB2 was significantly lower than that of ErbB3, and HRG induced a less pronounced decrease in the diffusion coefficient of all ErbB2 molecules and ErbB3/ErbB2 heterodimers than in the mobility of ErbB3. The slower diffusion of ErbB2 compared to ErbB3 was abolished by depolymerizing actin filaments, whereas ErbB2 expression induced a substantial rearrangement of microfilaments, implying a bidirectional interaction between ErbB2 and actin. HRG stimulation of cells co-expressing ErbB3 and ErbB2 led to the formation of ErbB3 homodimers and ErbB3/ErbB2 heterodimers in a competitive fashion. Although pertuzumab, an antibody binding to the dimerization arm of ErbB2, completely abolished the formation of constitutive and HRG-induced ErbB3/ErbB2 heterodimers, it only slightly blocked ErbB3 homodimerization. The results imply that a dynamic equilibrium exists between constitutive and ligand-induced homo- and heterodimers capable of shaping transmembrane signaling.

## Significance

ErbB3 is a growth factor receptor whose activation by its ligand, heregulin, leads to its homodimerization and heterodimerization with ErbB2. We applied two-color single molecule tracking and counting to quantitate the homo- and heterodimerization of ErbB3. Because of significant improvements in the applied method, introduced in the current manuscript, we show that ErbB3 is mostly monomeric in the absence of stimulation and ErbB2 co-expression. Both ligand stimulation and the presence of ErbB2 lead to significant retardation of ErbB3 lateral diffusion as well as increased formation of ErbB3 homodimers. Ligand stimulation in the presence of ErbB2 also induced heterodimers of ErbB3 and ErbB2. The results allow insight into the first steps of ErbB3 activation in a minimally perturbed system.

## Introduction

The four ErbB receptors (ErbB1–4) constitute a family of transmembrane proteins standing in the focus of interest of basic researchers and clinicians. Upon ligand-induced, overexpression- or mutation-driven activation of their intracellular kinase domain phosphorylated tyrosine residues are generated in their C-terminal part, leading to the activation of the mitogen-activated protein kinase (MAPK), phosphatidylinositol 3-kinase (PI3K), and signal transducer and activator of transcription (STAT) signaling pathways (1). Because transphosphorylation is responsible for the generation of phosphotyrosine residues, receptor clustering is required for activating these receptors. In the case of ErbB1, also known as epidermal growth factor (EGF) receptor, monomeric inactive receptors undergo ligand-induced dimerization accompanied by conformational changes in the extracellular, transmembrane, and intracellular kinase domains, culminating in the activation of the receptor (2, 3, 4). ErbB4 is also believed to work according to this above model (5). ErbB1 and ErbB4 can be considered to be full-fledged receptors for EGF-like and heregulin (HRG)-type ligands, respectively, because they contain fully functional ligand binding and tyrosine kinase domains (1). On the other hand, ErbB2 and ErbB3 harbor only half of the activity required for full activation, with ErbB2 lacking an activating soluble ligand and ErbB3 containing a not fully functional kinase domain (6). However, ErbB3/ErbB2 heterodimers formed upon binding of HRG to ErbB3 constitute the most potent oncogenic unit capable of strong activation of both the MAPK and PI3K pathways (7). The major role of ErbB2 is to enhance the potency and durability of transmembrane signaling by serving as the preferred heterodimerization partner for all other ErbB proteins (8).

Binding of its ligand, HRG, to ErbB3 induces the closed conformation of the receptor to adopt an extended structure in which a loop capable of promoting dimerization is exposed (9, 10). These structural changes are similar to what is observed after the binding of EGF to ErbB1 (3). The mechanism of ligand-induced ErbB3 activation beyond these first steps, however, is controversial. Both ErbB3 homodimerization and ErbB3/ErbB2 heterodimerization are believed to involve the dimerization arm, and therefore, the formation of these dimers must be mutually exclusive (11). Besides the back-to-back dimers promoted by the dimerization arm, ErbB1 has been shown to form several structurally unrelated clusters (12, 13), which may also exist for ErbB3. Large-scale homoclusters of ErbB2 and ErbB3 of undefined stoichiometry have been shown to be disrupted by HRG (14, 15, 16). The effect of ligand binding on ErbB3 homodimers is debated. Although HRG had no effect on the stability of ErbB3 homodimers according to chemical cross-linking experiments (14), results of single particle tracking implied that the growth factor stabilizes ErbB3 homodimers, which are signaling competent and even more stable than ErbB3/ErbB2 heterodimers (17). Although the HRG-induced stabilization of ErbB3/ErbB2 heterodimers is widely accepted (11, 17, 18), questions linger about the molecular mechanism of signal transduction by this heterodimer. The kinase domains are not equivalent in the dimer because the kinase of the ligand-bound receptor (“cis-kinase”) is assumed to adopt the receiver conformation and to be activated first by its dimerization partner (4, 19). According to this model, the ErbB3 kinase (“cis-kinase”) must be activated by ErbB2 in an ErbB3/ErbB2 heterodimer so that ErbB2 can be phosphorylated. Because ErbB3 is thought to be kinase dead (6), several suggestions have been put forward to resolve this contradiction: 1) the kinase domain of ErbB3 has been suggested to be conditionally active in the presence of ErbB2 (17, 20); 2) ErbB2 can also be activated by secondary dimerization (17, 21); and 3) tetramers or even larger-scale clusters can also provide a platform in which dimers can transactivate each other (13, 18).

Detailed biophysical studies revealed the existence of three hierarchical layers of receptor clusters: 1) dimerization driven by protein-protein interactions; 2) oligomerization generated by preferential distribution in lipid microdomains (referred to as large-scale clusters in the previous paragraph); and 3) clusters with a diameter of ∼100 nm or more visible even in light microscopy (22, 23). Biophysical techniques show different potentials to detect these clusters, depending on their size, stability, and mobility (22, 24).

The aim of the current investigation was to analyze homo- and heterointeractions of ErbB3 in living, minimally perturbed quiescent and HRG-activated cells. We used a single molecule imaging technique termed “thinning out clusters while conserving stoichiometry of labeling” (TOCCSL) (25) to quantitatively measure the mobility and dimerization of ErbB3. We define clusters from the standpoint of TOCCSL as long-lived and mobile entities. Our results show that the overwhelming majority of ErbB3 is monomeric in the absence of HRG stimulation and ErbB2 co-expression. Constitutive ErbB3 homodimers, accounting for ∼10% of ErbB3, exhibit retarded lateral diffusion, which is further reduced by HRG stimulation. In the presence of ErbB2 co-expression, ErbB3 undergoes HRG-induced homodimerization as well as heterodimerization with ErbB2. The lateral mobility of ErbB2 was significantly smaller than that of ErbB3, and it was less substantially affected by HRG. Pertuzumab (PRT), an antibody binding to the dimerization arm of ErbB2, blocked HRG-induced heterodimerization but inhibited the formation of ErbB3 homodimers only partially. The results show how a dynamic equilibrium established in homo- and heterodimerization events accompanies activation of ErbB3.

## Materials and Methods

### Cells

Chinese hamster ovary (CHO) cells were obtained from the American Type Culture Collection (Manassas, VA) and cultured according to its specifications. The CHO-ErbB2 cell line was generated as described previously (26), and it was continuously cultured in Dulbecco’s Modified Eagle Medium supplemented with 10% fetal calf serum and 20 *μ*g/mL G418. CHO-ErbB3 and CHO-ErbB2-ErbB3 cells were generated by transient transfection of the ErbB3-pcDNA6 plasmid using TurboFect Transfection Reagent (Thermo Fisher Scientific, Waltham, MA) into wild-type CHO and CHO-ErbB2 cells, respectively. To visualize actin filaments, cells were transfected with LifeAct-GFP (plasmid kindly provided by Miklós Kellermayer, Department of Biophysics and Radiation Biology, Semmelweis University, Budapest, Hungary) using TurboFect. LifeAct-GFP has been shown to be superior to actin-GFP for studying the dynamics and organization of microfilaments in live cells (27). For microscopy, cells were harvested with Accutase (Sigma-Aldrich, St. Louis, MO) and transferred to fibronectin-coated eight-well chambered coverglass (Thermo Scientific Nunc, Rochester, NY). Fibronectin-coating of coverslips was carried out by covering them with 125 *μ*L of a 50 *μ*g/mL fibronectin (Sigma-Aldrich) solution for 2 h, followed by removing the solution and drying. For flow cytometry, cells were harvested by trypsinization.

### Cloning

To generate the ErbB3-pcDNA construct, nontagged wild-type ErbB3 was amplified from the erbB3–pEGFP-N1 plasmid (kind gift of Donna Arndt-Jovin, Max Planck Institute for Biophysical Chemistry, Göttingen, Germany) with Phusion High-Fidelity DNA Polymerase (Thermo Fisher Scientific), according to the recommendations of the manufacturer. The primers used for the PCR reaction introduced a KpnI and a NotI cleavage site at the end of the amplified sequence (5′–ATCTGCGGTACCATGAGGGCGAACGACGCTCT, 5′–TATCGTGCGGCCGCTTACGTTCTCTGGGCATTA). The PCR product was isolated from agarose gel and cleaned up with NucleoSpin Gel and PCR Clean-up kit (Macherey-Nagel, Düren, Germany). Both the PCR product and the pcDNA6 (Thermo Fisher Scientific) plasmid was digested with KpnI (Thermo Fisher Scientific) and NotI (Thermo Fisher Scientific), and these DNA fragments were ligated with T4 DNA ligase (Thermo Fisher Scientific) as recommended by the manufacturer. Cloning was confirmed with restriction analysis and sequencing.

### Antibodies and chemicals

The monoclonal antibody H3.90.12 against ErbB3 was purchased from Thermo Fisher Scientific, whereas the anti-ErbB2 antibody 76.5 was produced from the supernatant of a hybridoma obtained from Yosef Yarden (The Weizmann Institute of Science, Rehovot, Israel). The isolated full-length antibodies were digested with papain to generate Fab fragments using the Pierce Fab Preparation Kit (Thermo Fisher Scientific). Papain, a nonspecific thiol-endopeptidase, enzymatically cleaves the whole immunoglobulin G (IgG) and creates two separate Fab fragments and one Fc fragment per antibody molecule. The reaction can be easily stopped by removing the resin from the IgG solution, and the result is an enzyme-free digestion product. Fab fragments were purified from partly digested or undigested full-length antibodies via gel filtration (Superdex 200, 10/300 GL; GE Healthcare Life Sciences) using the ÄKTA pure chromatography system (GE Healthcare Life Sciences, Pittsburgh, PA). Fab fragments were conjugated with Alexa Fluor 488 (AF488) and Alexa Fluor 647 (AF647, Thermo Fisher Scientific) using the N-Hydroxysuccinimide ester derivatives of the dyes according to the instructions of the manufacturer. The fluorescent dye-conjugated Fab fragments were separated from an excess of unreacted dye by means of gel filtration (Superdex 75, 10/300 GL; GE Healthcare Life Sciences). The protein-containing fractions were concentrated with Amicon Ultra-4 centrifugal filters (10 kDa cutoff; MilliporeSigma, Burlington, MA) to 1 mg/mL and stored in 50% glycerol and phosphate-buffered saline (PBS) at −20°C. The degrees of labeling of the fluorescent dye-conjugated Fab fragments were 1.5 (Fab 90.12-AF488), 1.8 (Fab 90.12-AF647), and 1.4 (Fab 76.5-AF488), as determined by spectrophotometry at 280 nm and at the corresponding absorption maximum of the fluorescent dye (488 and 647 nm).

The following antibodies were used in the tyrosine phosphorylation experiments: mouse monoclonal antibody PY99 against phosphotyrosine (Santa Cruz Biotechnology, Dallas, TX); rabbit monoclonal antibody ab133443 against ErbB3 phosphorylated at Y1289 (Abcam, Cambridge, UK); AF647 goat anti-mouse IgG; and AF647 goat anti-rabbit IgG (Thermo Fisher Scientific). The EGF domain of HRG-β1 was obtained from R&D Systems (Minneapolis, MN).

### Immunofluorescence measurements

Cells plated in fibronectin-coated, eight-well chambered coverglass 1 day before the measurement were washed in cold PBS and incubated at 37°C in the presence or absence of 200 ng/mL (25 nM) HRG followed by fixation in 3.7% formaldehyde for 30 min on ice. After washing and permeabilization with 0.1% (v/v) Triton X-100, cells were labeled with antibodies against phosphotyrosine or tyrosine phosphorylated ErbB3 for 30 min on ice followed by washing and secondary staining with AF647-conjugated goat anti-mouse IgG or with AF647-conjugated goat anti-rabbit IgG for 30 min on ice. Finally, the samples were washed in PBS followed by fixation in 1% formaldehyde. Images were acquired with a Zeiss LSM 700 confocal microscope (Carl Zeiss, Oberkochen, Germany) using a 63× oil immersion objective (NA = 1.35). AF488 was excited at 488 nm, and its fluorescence was detected above 510 nm. AF647 was excited at 633 nm, and its fluorescence was collected above 670 nm.

### Labeling and treatment of cells for TOCCSL and FRAP experiments

Cells were plated in fibronectin-coated, eight-well chambered coverglass 30 min before the measurement. The cells were washed in PBS and labeled with a mixture of AF488- and AF647-conjugated Fab fragments at an equimolar ratio (10–10 *μ*g/mL). They were incubated at room temperature for 15 min. To remove unbound fluorescent probes, the cells were washed twice in PBS, followed by incubation with or without 200 ng/mL (25 nM) HRG. This labeling protocol led to homogeneous surface staining for both colors.

### Determination of the dissociation constant of Fab fragments

For cell surface labeling, 0.25–1 × 10^6^ CHO-ErbB2-ErbB3 cells were stained with AF488-conjugated Fabs for 30 min on ice and washed three times with FACS buffer (PBS, 1% bovine serum albumin, and 0.02% NaN_3_). The dissociation constants (*K*_d_) of AF488-conjugated and unlabeled Fabs were measured by a Becton Dickinson LSR II flow cytometer (BD Biosciences, Franklin Lakes, NJ). Despite labeling on ice, AF647-coupled anti-ErbB3 Fab accumulated intracellularly, preventing an accurate measurement of membrane-bound intensity by flow cytometry. Therefore, CHO-ErbB2-ErbB3 cells were labeled with the AF647-conjugated Fab in chambered coverglass, and they were imaged with a Zeiss LSM 700 confocal microscope as described above. Confocal microscopic images were analyzed with the DIPimage toolbox (Delft University of Technology, Delft, the Netherlands) under MATLAB (The Mathworks, Natick, MA). The cell membrane was identified by a manually seeded watershed algorithm (28, 29) using a custom-written interactive algorithm implemented in DIPimage/MATLAB. The fluorescence intensity was evaluated in the membrane mask determined by manually seeded watershed segmentation after subtracting the background determined in a cell-free area of an image. The mean fluorescence intensity of flow cytometric histograms of cells labeled with AF488-conjugated Fab or the mean membrane intensity of cells labeled with AF647-coupled Fab were used for further analysis. For determining the *K*_d_ of labeled Fabs, equilibrium binding of a concentration series of fluorophore-conjugated Fabs was measured. To determine the *K*_d_ of unlabeled Fabs, cells were labeled with a concentration series of unlabeled Fab in the presence of a constant concentration of labeled Fab. The mean fluorescence intensity of samples was determined after gating out cell fragments and debris on the forward and side scatter dot plot in ReFlex, a flow cytometry evaluation program (30). To determine the *K*_d_ of AF488- and AF647-conjugated Fab fragments, we measured the equilibrium binding of a concentration series of the corresponding Fab fragments. The *K*_d_ of fluorescent Fabs was determined by fitting a one-site, specific binding model to the measured data points. To determine the *K*_d_ of unlabeled Fab fragments, cells were labeled with a concentration series of unlabeled Fab in the presence of a constant concentration of AF488- or AF647-conjugated Fab fragments. The *K*_d_ of unlabeled Fabs was calculated by fitting a one-site, competitive binding model to the data points.

### Single molecule microscopy

**Figure 1:**
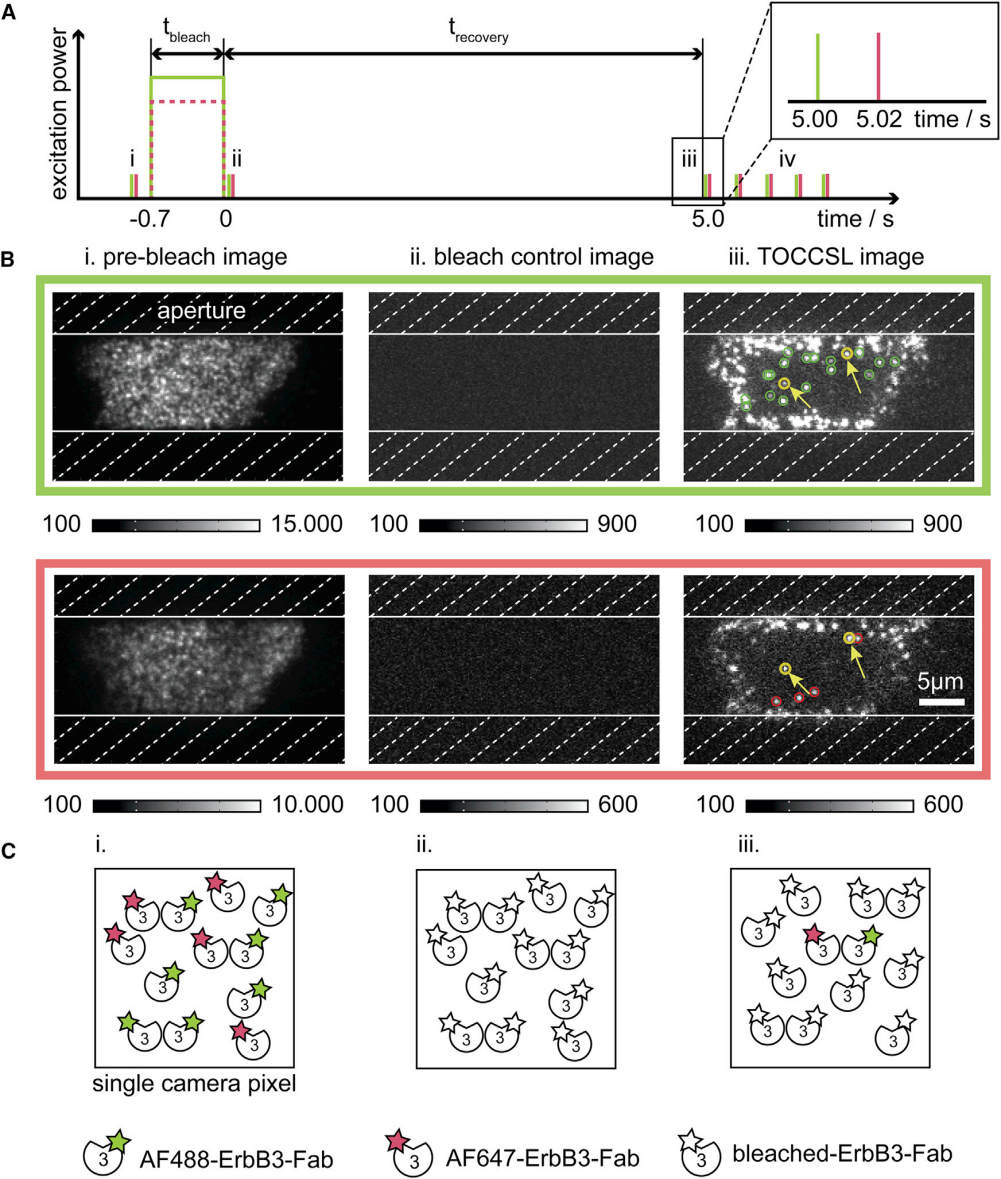
Experimental strategy for detection of ErbB3 dimerization. (*A*) The laser power/pulse duration and image acquisition protocol applied in two-color TOCCSL experiments is indicated. The inset shows a zoom into the timing protocol with a delay time of 20 ms between recordings of the green and the red color channel. (*B*) ErbB3-positive CHO cells growing on fibronectin coated coverslips were quantitatively decorated with equimolar amounts of AF488- and AF647-labeled monovalent antibody fragments (AF488-ErbB3 Fab and AF647-ErbB3 Fab). A defined CHO cell region was illuminated in TIR mode by using an aperture (*dashed area*) in the excitation pathway. Emission was split into two-color channels and imaged on the same electron-multiplying charge-coupled device camera. The average ErbB3 surface density was too high to allow for the resolution of single entities (*i*), yielding many ErbB3 molecules per camera pixel (see sketch below). After applying a 0.7-s long photobleaching pulse, all fluorophores were ablated as can be seen in the image recorded 10 ms after bleaching (*ii*). At the onset of the recovery process after 5–10 s, single probes that have diffused from the masked region into the central field of view can be seen as diffraction limited signals (*iii*). Single diffraction limited signals were detected in both color channels (*green* and *red circles*), and homoassociation was determined by the co-localization of signals within a radius of 160 nm (*yellow circles*). After the TOCCSL image, an additional image sequence was recorded and allowed for tracking of co-localized and individual ErbB3 signals for the subsequent determination of diffusion constants (laser pulses *iv* in the *upper* panel). (*C*) A sketch showing the effect of the photobleaching protocol on putative ErbB3 homodimers is shown. To see this figure in color, go online.

Single molecule experiments were performed as described (31, 32). Briefly, an Axiovert 200 microscope equipped with a 100× NA = 1.46 Plan-Apochromat objective (Carl Zeiss) was used for illuminating samples in objective-based total internal reflection (TIR) configuration via the epiport by 488-nm (Sapphire; Coherent, Santa Clara, CA) or 640-nm (iBeam SMART; TOPTICA Photonics, Munich, Germany) laser light with a typical power of 0.5–5 kW/cm^2^ on the sample. The experiments were carried out at room temperature to slow down the dissociation of Fabs. For exact timing of the 488-nm laser, the beam path was equipped with an acousto-optic modulator (Isomet, Manassas, VA). A slit aperture (Thorlabs, Newton, NJ) with a width of ∼10 *μ*m in the sample plane was used as a field stop. An in-house written program package implemented in LABVIEW together with a high-speed analog output card (National Instruments, Austin, TX) were used to generate timing protocols. Emission light was separated from excitation by a dichroic mirror (zt488/640 rpc; Chroma Technology, Bellows Falls, VT), split into two-color channels equipped with appropriate filters (HQ585/40m, HQ700/75m; Chroma) using a dual-view system (Photometrix, Kew, Australia), and imaged on a back-illuminated electron-multiplying charge-coupled device camera (iXon Ultra 897; Andor Technology, Belfast, UK). An exemplary laser intensity and pulse duration protocol is provided in Fig. 1. After recording prebleach images with a power density of 0.5 kW/cm^2^ and an illumination time of t_ill_ = 5 ms (Fig. 1 *i*), samples were bleached with a laser pulse applied for 700 ms with a power density of 5 kW/cm^2^. After a recovery time of 5–10 s, TOCCSL images were recorded with the same settings as for the prebleach images (Fig. 1 *iii*). Photobleaching was checked by recording images 10 ms after the bleach pulse (Fig. 1 *ii*). Although the bleach pulses for both color channels were applied simultaneously, the prebleach and TOCCSL images were recorded with 20-ms time gaps between the two channels.

### Data analysis in single molecule microscopy

A detailed description of the applied co-localization analysis has been provided previously (32). For precise alignment of both color channels, fluorescent multicolor beads (TetraSpeck; Thermo Fisher Scientific) were immobilized on coverslips and imaged under conditions identical to those used for TOCCSL experiments. Positions of beads in both color channels were determined with custom-written MATLAB scripts based on a maximal-likelihood estimator and used to calculate the relative shift and stretch of the two-color channels with respect to each other. In general, positions of the same bead imaged in two colors were aligned with accuracies down to ∼20 nm, which can be attributed to the correction error of the method because localization errors were found to be smaller than 5 nm. For TOCCSL data on living cells, single molecule positions were determined and corrected, and positions found in both color channels within a distance of one camera pixel (i.e., 160 nm) were counted as co-localized. For co-localization events, virtual distances between AF488-ErbB3-Fab and AF647-ErbB3-Fab signals were determined. The number of false-positive co-localizations was determined by mirroring the coordinates of one color channel alongside the *x* and *y* axis through the center of mass of all signals before applying the search algorithm for co-localization. Co-localization fractions were calculated by counting all co-localized signals, subtracting false positives, correcting for nonequimolar labeling and for the presence of unlabeled ErbB3 (i.e., ErbB3 bound by an unlabeled Fab), and correcting for the difference in diffusion coefficients, as outlined in the Supporting Materials and Methods. Single molecule tracking was performed as described previously (33). A *msd* versus time-lag analysis was used to determine the diffusion coefficient. Mean-squared displacements (*msd*) were calculated from all trajectories of all cells for a certain condition and plotted as a function of time lags (*t*_lag_). Using the first two data points, the diffusion was calculated by using the formula *msd* = 4 *D t*_lag_ + 4 *σ*_xy_^2^, where *σ*_xy_ denotes the localization precision. As described elsewhere, using only the first two data points provides the best estimate of the diffusion coefficient (33). For the determination of the error of the diffusion coefficient, a sampling approach was used. Random numbers for *msd* values were drawn from a Gaussian-shaped distribution (with a mean equal to the *msd*, and a SD identical to the SD of squared displacements for the respective time lag) for the first two time lags, and the diffusion coefficient was calculated. This procedure was repeated 1000 times, and the SD, representing the reliability of fitting, was determined.

### FRAP experiments

FRAP experiments were carried out similarly to what was described in the Single molecule microscopy. To avoid bleaching of dyes during the recovery phase, the laser power for the pre- and postbleach images was further reduced to ∼30 W/cm^2^. After the bleach pulse, up to 120 postbleach images were recorded with a time lag of t_lag_ = 2 s. For correcting photobleaching during image acquisition, fast image sequences with t_lag_ = 15 ms were recorded. For calculation of the photobleaching rate, *k*_*bleach*_, the background-corrected brightness values for all images, *I(i)*, were determined with FIJI, and an exponential decay curve was fitted in MATLAB as follows:

(1) $$\text{I}\left(\text{i}\right)={\text{I}}_{0}\left({\text{e}}^{-\text{i}\cdot {\text{k}}_{\text{bleach}}}\right),$$

with I_0_ as the initial brightness.

FRAP data was analyzed with software packages implemented in MATLAB corrected for photobleaching, and a nonlinear least-square fit was used to determine the mobile fraction, f_m_, as follows:

(2) $$\frac{\text{I}\left(\text{i}\cdot {\text{t}}_{\text{lag}}\right)}{{\text{I}}_{\text{pre}}}={\text{f}}_{\text{m}}\left(1-{\text{e}}^{-\frac{\text{i}\cdot {\text{t}}_{\text{lag}}}{\tau }}\right),$$

where *I(i⋅t*_*lag*_*)* is the photobleaching and background-corrected brightness in image *i*, *I*_*pre*_ is the brightness of the prebleach image, and *τ* is the characteristic constant for fluorescence recovery.

Each recorded cell was analyzed individually before data were pooled to calculate the mean and SD of mobile fractions.

### Airyscan microscopy

The cell culture solution on cells plated on eight-well chambered coverglass 1 day before the measurement was replaced by Hank’s balanced salt solution supplemented with 10 mM glucose. Imaging was performed using the “superresolution” mode of the Airyscan module of a Zeiss LSM880 confocal microscope (Carl Zeiss) at 37°C. LifeAct-GFP was excited at 488 nm, and its emission was collected between 495 and 620 nm. Image stacks were obtained with a pixel size of 42 nm and a separation distance between consecutive slices of 300 nm using a 40× water immersion objective (NA = 1.2).

## Results

### HRG-driven homodimerization of ErbB3 in the absence of ErbB2 co-expression

We selected CHO cells for all experiments because they do not express ErbB proteins (8). Cells were transiently transfected with human ErbB3 and responded to HRG stimulation in accordance with the previous results (8) (i.e., HRG did only increase tyrosine phosphorylation of ErbB3 in the presence of ErbB2 co-expression) (Fig. S1).

For single molecule experiments, CHO cells were seeded onto fibronectin-coated glass coverslips and allowed to adhere before they were labeled with a mixture of Alexa Fluor 488-coupled (AF488) and Alexa Fluor 647-coupled (AF647) Fab fragments against ErbB3 (AF488-ErbB3-Fab and AF647-ErbB3-Fab). The concentration ratio of the two Fabs was chosen in accordance with their determined affinities for ErbB3 (Table S1) to achieve similar labeling efficiency of the two colors. The high ErbB3 density at the plasma membrane prevented a direct assessment of ErbB3 oligomerization, so we utilized TOCCSL to stoichiometrically reduce the surface density down to a level in which single molecules can be observed as well-separated spots (25, 31, 34). Fig. 1 shows the principle of two-color TOCCSL (32). For estimating the surface density of both labels, we first recorded a prebleach image in both colors using an epifluorescence microscope configured in TIR mode (Fig. 1 *i*). Next, a short, two-color high-power laser pulse was used to photobleach all active fluorophores within a small spatially defined area on the plasma membrane facing the coverslip. We verified complete photobleaching by recording a second image immediately after bleaching (Fig. 1 *ii*). During the recovery phase, fluorescently labeled ErbB3 receptors diffused from the masked cell surface back into the field of view and had not undergone any bleaching. At the onset of this process (here, between 5 and 10 s), the density of fluorescent molecules was low enough to detect them as well-separated diffraction limited signals in the so-termed TOCCSL image (Fig. 1 *iii*, *green* and *red circles*). Single molecule events corresponding to co-localized AF488- and AF647-ErbB3-Fabs could be identified via the registration of both color channels and are referred to as ErbB3 homodimers (Fig. 1 *iii*, *yellow circles*). Consecutive recording of an image sequence allowed for the tracking of individual signals visible in both color channels and of co-localized ErbB3 signals (Fig. 1 *iv*).

For the first species, corresponding to all recovered ErbB3 signals, we determined a diffusion coefficient of 0.084 ± 0.005 *μ*m^2^/s (see Table 1 for a list of determined diffusion constants). This value can be regarded also as the diffusion constant of ErbB3 monomers because this is the dominant species contributing the most to tracking data. If the analysis was restricted to co-localized ErbB3 spots, the diffusion constant was 0.052 ± 0.022 *μ*m^2^/s, suggesting that homodimers of ErbB3 move more slowly.

**Table 1:** Diffusion Constants of Monomeric and Dimeric Species of ErbB3

	Molecule Species	Cell Line (Fluorescent Labels)	HRG	PRT	Diffusion Coefficient *μ*m^2^/s, ±SD (n)
Homodimerization experiment	All ErbB3	CHO-ErbB3 (AF488-ErbB3-Fab, AF647-ErbB3-Fab)	−	−	0.084 ± 0.005 (1991)
	Co-localized ErbB3		−	−	**0.052 ± 0.022 (81)**
	All ErbB3		+	−	0.053 ± 0.004 (1657)
	Co-localized ErbB3		**+**	−	**0.014 ± 0.02 (85)**
	All ErbB3	CHO-ErbB2-ErbB3 (AF488-ErbB3-Fab, A647-ErbB3-Fab)	−	−	0.095 ± 0.005 (7351)
	Co-localized ErbB3		−	−	**0.031 ± 0.06 (62)**
	All ErbB3		+	−	0.056 ± 0.002 (9496)
	Co-localized ErbB3		**+**	−	**0.018 ± 0.006 (322)**
	All ErbB3		−	+	0.053 ± 0.004 (3195)
	Co-localized ErbB3		−	**+**	**0.043 ± 0.012 (95)**
	All ErbB3		+	+	0.026 ± 0.004 (3050)
	Co-localized ErbB3		**+**	**+**	**0.017 ± 0.007 (136)**
Heterodimerization experiment	All ErbB2	CHO-ErbB2-ErbB3 (AF488-ErbB2-Fab, A647-ErbB3-Fab)	−	−	0.044 ± 0.003 (1842)
	All ErbB3		−	−	0.067 ± 0.006 (789)
	Co-localized ErbB3/ErbB2		−	−	**0.017 ± 0.006 (80)**
	All ErbB2		+	−	0.034 ± 0.002 (3394)
	All ErbB3		+	−	0.040 ± 0.008 (390)
	Co-localized ErbB3/ErbB2		**+**	−	**0.010 ± 0.007 (26)**
	All ErbB2		−	+	0.018 ± 0.002 (2791)
	All ErbB3		−	+	0.054 ± 0.006 (749)
	Co-localized ErbB3/ErbB2		−	**+**	**0.022 ± 0.008 (82)**
	All ErbB2		+	+	0.029 ± 0.003 (2358)
	All ErbB3		+	+	0.065 ± 0.006 (725)
	Co-localized ErbB3/ErbB2		**+**	**+**	**0.026** ± **0.017 (42)**

Untreated, HRG-, and/or PRT-pretreated CHO cells expressing ErbB3 in the absence (CHO-ErbB3) or presence (CHO-ErbB2-ErbB3) of ErbB2 were labeled with AF488- and AF647-conjugated Fabs as shown in the table. The mean diffusion coefficients (±SD) were determined by tracking individual fluorescent spots. Error estimation was carried out using a sampling approach as described in Materials and Methods. The number of trajectories (n) analyzed is also given in the table. Rows labeled by headings “All ErbB2” or “All ErbB3” display data for all fluorescent spots of a certain color (e.g., a mixture of monomers and dimers). Because of the overwhelming majority of monomeric events among trajectories, these values correspond to the diffusion coefficients of monomers. The analysis was restricted to co-localized signals in the rows labeled by “Co-localized ErbB3” and “Co-localized ErbB3/ErbB2.”

**Figure 2:**
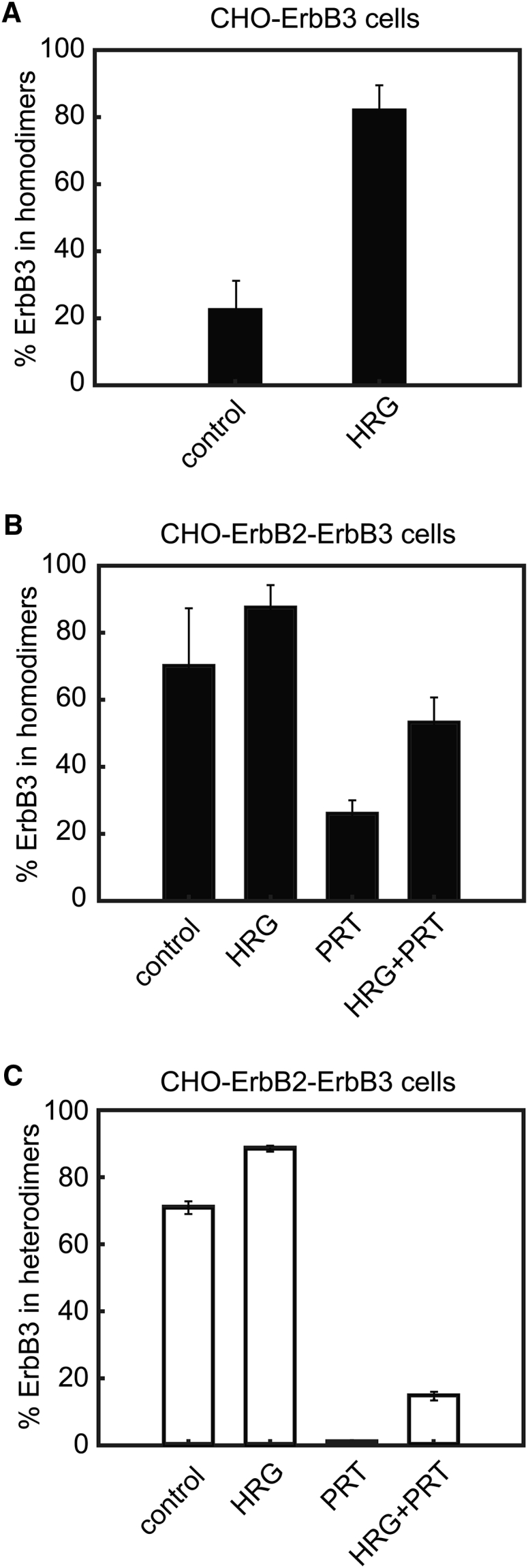
The fraction of ErbB3 associated to homo- and heterodimers in CHO-ErbB3 and CHO-ErbB2-ErbB3 cells determined by two-color TOCCSL. The mean (± standard error of the mean) of the homo- (*black bars*) (*A* and *B*) and heterodimeric fractions (*white bars*) (*C*) of ErbB3 are shown under different treatment conditions (HRG, heregulin; PRT, pertuzumab). In TOCCSL recordings, co-localized signals were counted, the respective numbers were corrected for labeling and for the different diffusion constants of monomers and dimers, and finally, the fraction of ErbB3 in dimers was calculated (see Supporting Materials and Methods for details of the correction). The corrected values, shown in the figure, and the raw data can be found in Table S2.

Because TOCCSL is based on the recovery of fluorescent signals due to the diffusion of unbleached membrane components into the photobleached region, different diffusion coefficients of monomers and dimers influence the fractions of these species observed in the bleached area. We developed a framework in which the different diffusion coefficients of monomers and dimers are explicitly considered and determined the corrected amount of ErbB3 monomers and homodimers. We opted to neglect larger oligomers for the following reasons: 1) in single-color TOCCSL experiments, taking the brightness of individual mobile clusters into consideration, monomers and dimers accounted for ∼90% of events (Fig. S2); and 2) because of cytoskeletal anchoring and induced membrane curvature, the apparent diffusion coefficient of larger clusters is expected to be much smaller than those of monomers and dimers, preventing their return to the bleached area (35). Our analysis yielded that ∼23% of all ErbB3 present on the membrane of CHO cells was associated to constitutive homodimers (Fig. 2 *A*).

We next addressed ErbB3 homoassociation under activating conditions. Cells were treated with HRG, and two-color TOCCSL experiments were performed in the presence of HRG. Interestingly, the fraction of ErbB3 in homodimers increased ∼4-fold (Fig. 2 *A*). Statistical significance of all comparisons is shown in Table S3. Tracking of all ErbB3 signals after HRG stimulation yielded a similar mobility as for ErbB3 homodimers under nonstimulatory conditions (0.053 ± 0.004 *μ*m^2^/s). The diffusion constant of ErbB3 homodimers was found to be reduced to 0.014 ± 0.02 *μ*m^2^/s (Table 1). Taken together, HRG strongly increases the fraction of stable ErbB3 homoassociates at the plasma membrane, accompanied by a reduction of their overall mobility.

### Homodimerization of ErbB3 in the presence of ErbB2 co-expression

To examine the effect of ErbB2 on the constitutive and ligand-induced homodimerization of ErbB3, CHO-ErbB2 cells, stably expressing ErbB2 at 200,000 ± 15,000 proteins/cell according to flow cytometry, were transiently transfected with ErbB3. Two-color TOCCSL experiments were performed as described before to determine the fraction of ErbB3 present in homodimers and to determine the respective diffusion coefficients. It turned out that in the presence of ErbB2, ∼70% of ErbB3 was associated to homodimers (Fig. 2 *B*). Although the mere presence of unlabeled ErbB2 elevated the base level of ErbB3 homodimers close to the level seen under stimulating conditions in the absence of ErbB2, stimulation with HRG further increased the fraction of ErbB3 in homodimers up to ∼90% (Fig. 2 *B*). Tracking of ErbB3 showed that the presence of ErbB2 did not substantially alter the mobility of all recovered ErbB3 molecules; however, the diffusion coefficient of ErbB3 homodimers was drastically reduced to one-third of the initial value compared to all ErbB3 molecules (Table 1). The diffusion coefficients of all ErbB3 molecules and ErbB3 homodimers after HRG stimulation was reduced to a similar extent to what was observed in the absence of ErbB2 co-expression (Table 1).

To test for a direct effect of the presence of ErbB2 on ErbB3 homodimerization, we repeated two-color TOCCSL experiments with cells treated with PRT, an antibody binding to the dimerization arm of ErbB2 (36, 37). Indeed, PRT reduced the fraction of ErbB3 in homodimers in the absence of stimulation down to ∼26% (Fig. 2 *B*; Table S3). In addition, the antibody had a similar inhibitory effect on the HRG-induced homodimerization (Fig. 2 *B*).

Our data hence show that the presence of ErbB2 directly affects the amount of ErbB3 molecules associated to homodimers as well as retards the diffusion of ErbB3 homodimers in the plasma membrane under both HRG-stimulated and nonactivating conditions.

### Heterodimerization of ErbB3 with ErbB2 in the absence and presence of HRG stimulation

**Figure 3:**
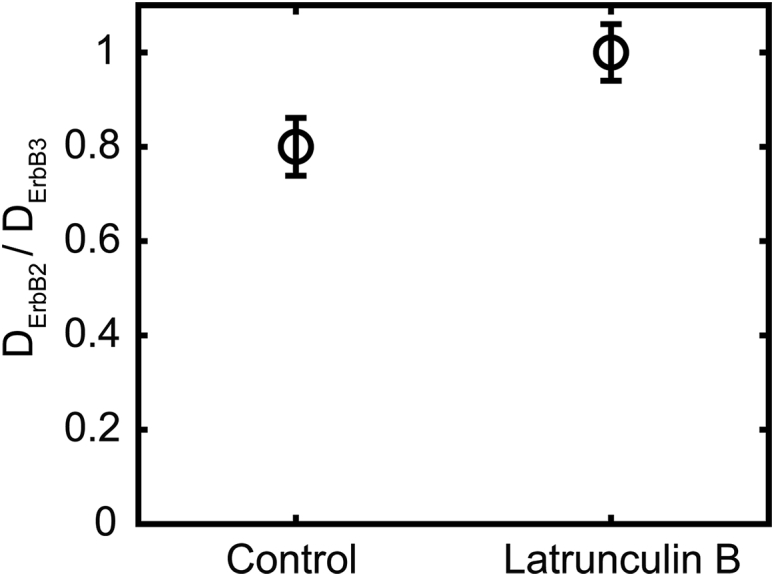
Disruption of actin filaments abolishes the difference between the diffusion coefficients of ErbB2 and ErbB3. CHO-ErbB2-ErbB3 cells were treated with 1 *μ*M latrunculin B for 10 min followed by labeling these treated and control cells with fluorescent anti-ErbB2 and anti-ErbB3 Fab at an equimolar ratio (10–10 *μ*g/mL). The ratio of the diffusion coefficients of ErbB2 and ErbB3 are plotted along with their standard error of the means.

CHO-ErbB2 cells transiently transfected with ErbB3 were labeled with AF488-ErbB2-Fab and AF647-ErbB3-Fab to measure the formation of ErbB3/ErbB2 heterodimers in two-color TOCCSL experiments as well as the diffusion coefficients of heterodimers and individual ErbB2 and ErbB3 molecules. According to the surface densities determined in the prebleach image of TOCCSL recordings, cells with similar ErbB2 and ErbB3 expressions were selected. We first determined the fraction of ErbB3 co-localizing with ErbB2 in the absence of HRG stimulation (see Supporting Materials and Methods for details of the correction and Table S2 for all raw and corrected data) and found a rather high base level of ∼70% of ErbB3 being associated to ErbB3/ErbB2 heterodimers (Fig. 2 *C*). Stimulation of CHO-ErbB2 cells with HRG led to a further increase of ErbB3/ErbB2 heterodimerization (∼90%, Fig. 2 *C*). Our tracking data revealed that ErbB2 had a twofold lower diffusion coefficient than ErbB3 in the absence of stimulation (Table 1). Given that the overwhelming majority of tracked events correspond to monomers (Fig. S2), this difference implies a fundamental difference between the diffusion of ErbB2 and ErbB3. The analysis of step lengths of tracked ErbB2 and ErbB3 events revealed that the distribution of the diffusion rate of ErbB2 is more variable, spanning a wider range of values (Fig. S3). Because actin filaments were assumed to be behind the aforementioned difference, the diffusional behavior of ErbB2 and ErbB3 was also compared in latrunculin B-treated cells, in which actin filaments were depolymerized. Although the difference between the diffusion coefficients of ErbB2 and ErbB3 was smaller in control cells in these experiments, the latrunculin B treatment completely abolished the difference between ErbB2 and ErbB3 due to eliminating actin-related retardation of ErbB2 (Fig. 3; Fig. S3). Cortical actin filaments exert a much smaller effect on ErbB3 than on ErbB2, evidenced by the lack of a significant effect of latrunculin B on ErbB3 diffusion. HRG treatment only slightly reduced the diffusion coefficient of ErbB2 from 0.044 ± 0.003 to 0.034 ± 0.002 *μ*m^2^/s. The difference in diffusion constants between the HRG-induced and constitutive ErbB3/ErbB2 heterodimers (0.01 ± 0.007 *μ*m^2^/s vs. 0.017 ± 0.007 *μ*m^2^/s) was not as pronounced as for ErbB3 homo-oligomerization.

We next wanted to test whether the dimerization arm of ErbB2 is involved in the formation of the observed ErbB3/ErbB2 heterodimers. We hence incubated the cells with PRT, which practically eliminated heterodimers in unstimulated cells and significantly reduced the HRG-induced formation thereof (Fig. 2 *C*).

In summary, both constitutive and HRG-induced ErbB3/ErbB2 heterodimers are present at high levels. Heterodimers diffuse more slowly than ErbB3 homodimers and are incapable of formation upon blockage of the dimerization arm of ErbB2.

### Effect of ErbB2 expression on actin filaments

**Figure 4:**
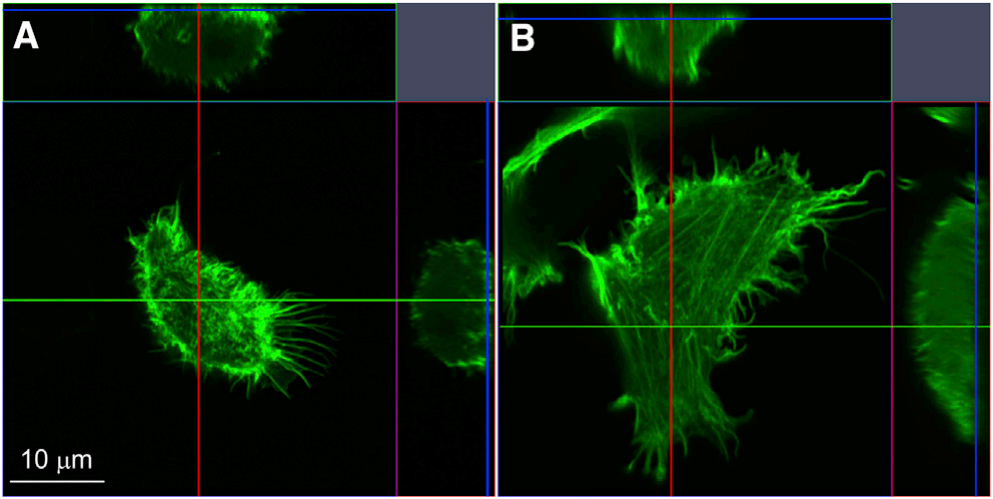
Airyscanning reveals significant rearrangement of actin filaments as a result of ErbB2 expression. Control (*A*) and ErbB2-expressing (*B*) CHO cells were transfected with LifeAct-GFP followed by Airyscanning. Representative orthogonal sections reveal a striking effect of ErbB2 on the arrangement of subcortical actin filaments as evidenced by the formation of long actin stress fibers. Image stacks have been made available on Mendeley (https://doi.org/10.17632/yxjb58nwr3.1). To see this figure in color, go online.

The slower diffusion of ErbB2 compared to ErbB3 turned out to be attributable to an intact actin cytoskeleton. Next, we tested whether ErbB2 expression exerts any kind of influence on the organization of microfilaments. ErbB2-expressing and control cells were transfected with GFP-LifeAct followed by imaging with Airyscanning confocal microscopy. Whereas ErbB2-negative cells exhibited a patchy distribution of actin in the image corresponding to the submembrane plane, ErbB2-expressing cells were rich in long actin stress fibers completely absent from control cells. Representative images are shown in Fig. 4. These results establish that ErbB2 and actin filaments exert a reciprocal effect on each other.

### Competition between the formation of ErbB3 homodimers and ErbB3/ErbB2 heterodimers

**Figure 5:**
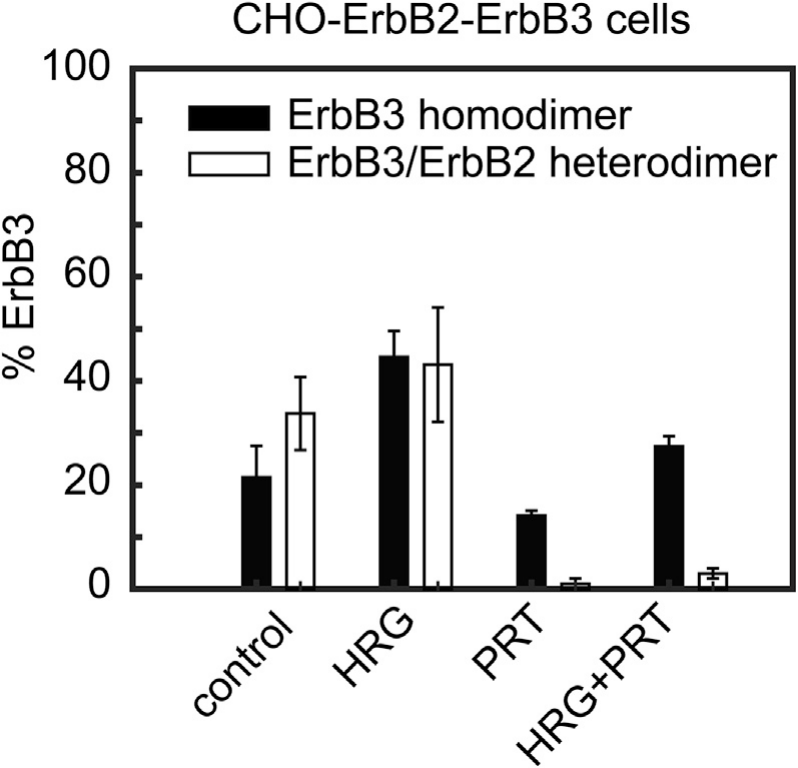
Competition between the association of ErbB3 to homo- and heterodimers. The corrected mean (± standard error of the mean) of the homo- (*black bars*) and heterodimeric fractions (*white bars*) of ErbB3 are shown under different treatment conditions (HRG, heregulin; PRT, pertuzumab). ErbB3 homo- and ErbB3/ErbB2 heterodimerization in CHO-ErbB2-ErbB3 cells was determined in independent experiments and analyzed based on a mathematical model accounting for the presence of ErbB3 homodimers, ErbB3/ErbB2 heterodimers, and ErbB3 monomers.

Utilizing CHO-ErbB2 cells and the above described two-color TOCCSL strategies allow only for the separate determination of ErbB3 homodimers and ErbB3/ErbB2 heterodimers. Whereas in the first set of experiments, ErbB3/ErbB2 heterodimers are missed, ErbB3 homodimers cannot be distinguished from single ErbB3 molecules in the second set. We hence developed a mathematical model that allowed for a global analysis of all data recorded in separate ErbB3 homodimer and ErbB3/ErbB2 heterodimer experiments (see Supporting Materials and Methods for details). ErbB2 co-expression reduced the fraction of ErbB3 in HRG-induced homodimers from ∼80% (Fig. 2 *A*) to ∼45% (Fig. 5), implying that competition takes place between the formation of ErbB3 homodimers and ErbB3/ErbB2 heterodimers. HRG stimulation led to a higher fold increase of ErbB3 being recruited to homodimers than to ErbB3/ErbB2 heterodimers. Treatment with PRT lowered the level of ErbB3 in homodimers for both conditions and nearly abolished the association of ErbB3 to ErbB3/ErbB2 heterodimers.

### Fluorescence recovery after photobleaching (FRAP) reveals immobilization of ErbB2 and ErbB3 after HRG stimulation

Given the fact that in TOCCSL experiments, only information about mobile species of ErbB2 and ErbB3 is gained and information on immobile molecules is absent, we were interested in the fraction of molecules probed by our approach. A conventional FRAP experiment allowed us to determine the mobile fraction of fluorescently labeled ErbB2 and ErbB3 under different treatment conditions. According to these measurements, ∼60% of ErbB3 was mobile in the absence of stimulation and ErbB2 co-expression (Table 2). Co-expression of ErbB2, in the absence of stimulation, decreased the mobile fraction of ErbB3 to ∼40% (Table 2). Interestingly, HRG stimulation of ErbB3 in the absence of ErbB2 co-expression had an effect similar to that of ErbB2 co-expression in that the growth factor reduced the mobile fraction of ErbB3 to ∼40%. Stimulation with HRG in the presence of ErbB2 co-expression almost completely immobilized ErbB3, with the mobile fraction being reduced to ∼17%. We next addressed the mobile fraction of ErbB2. We found that it was similar to that of ErbB3 and not influenced by HRG stimulation (Table 2). Although the diffusion coefficients of monomeric and dimeric species differed substantially, the low fraction of dimers precluded the observation of a multiexponential FRAP recovery curve. In summary, both HRG stimulation and ErbB2 co-expression decrease the mobile fraction of ErbB3.

**Table 2:** Mobile Fraction of ErbB2 and ErbB3 in the Absence and Presence of HRG Stimulation

Mobile fraction	CHO-ErbB3	CHO-ErbB2-ErbB3
ErbB3	0.62 ± 0.09	0.41 ± 0.11
ErbB3 + HRG	0.40 ± 0.10	0.17 ± 0.06
ErbB2	–	0.55 ± 0.15
ErbB2 + HRG	–	0.55 ± 0.18

The mean (±SD) of the mobile fractions of ErbB2 and ErbB3 were determined in CHO-ErbB3 and CHO-ErbB2-ErbB3 cells before and after HRG stimulation.

## Discussion

In the presented study, we tracked single, mobile, fluorescently labeled ErbB2 and ErbB3 proteins in living cells to measure their constitutive and HRG-induced dimerization, which led to the following major findings: 1) ErbB3 is mostly monomeric in the absence of stimulation and ErbB2 co-expression; 2) the diffusion coefficient of constitutive homodimers of ErbB3 is significantly lower than that of monomers; 3) HRG induces both ErbB3 homodimers and ErbB3/ErbB2 heterodimers, which compete with each other; 4) these homo- and heterodimers differ in terms of their sensitivity to inhibition by PRT; 5) the lateral diffusion of ErbB2 is slower than that of ErbB3, which is attributable to intact microfilaments; 6) ErbB2 is retarded less significantly by HRG stimulation than ErbB3; and 7) ErbB2 facilitates the formation of ErbB3 homodimers and retards the lateral mobility of these homodimers.

The single molecule technique TOCCSL used in our investigations detects mobile entities capable of returning to the photobleached area. Although this shortcoming prevents us from detecting immobile clusters, the combination of diffusion measurements, detection of co-localizations and the global analysis of homo- and heterodimerization experiments allowed us to improve the reliability of TOCCSL measurements significantly: 1) dimer fractions were corrected by considering the slower lateral mobility of dimers compared to monomers; and 2) although homodimerization of ErbB3 was investigated independently of its heterodimerization with ErbB2, results of these experiments were analyzed together, permitting us to observe their competitive formation upon HRG stimulation. Although we made every effort to compensate for the lower mobility of clustered proteins, trimers or even larger clusters of ErbB3, suggested previously (15, 18), may have escaped detection because of their expected very slow diffusion and have not been considered for any mathematical model in our study. Tracking methods typically involve some kind of undersampling. In TOCCSL, it is manifested in the undersampling of low-mobility species, which is taken into consideration, as described in Fig. S4. Tracking of Qdots, applied to studying ErbB proteins, involves significant underlabeling of receptors (17, 38). Although these features set certain limits on the sensitivity of these methods, mathematical corrections can shed light on the undersampled fraction of receptors.

The diffusion coefficients obtained in the current experiments using Fab labeling of ErbB2 and ErbB3 were larger by a factor of two than those reported previously using Qdot labeling (17). Qdots have been shown to induce a 35-fold reduction in the mobility of B cell receptors compared to Fab labeling, revealing a significant effect of bulky labels (39). The absence of an orders of magnitude difference between the diffusion coefficients of ErbB proteins reported in the current manuscript and those found with Qdot labeling by Steinkamp et al. is explained by the fact that our experiments were performed at room temperature to reduce the dissociation of Fabs, whereas the experiments with Qdot-labeled ErbB3 were performed at 37°C. From the comparison of these diffusion coefficients, it seems that the room temperature-induced retarded diffusion of Fab-labeled proteins is counterbalanced by the Qdot-dependent hindering of mobility. The PRT-induced reduction in ErbB2 diffusion can also be attributed to the bulky antibody. However, retardation of the mobility of ErbB3 homodimers by coexpressed ErbB2 merits further consideration because TOCCSL cannot distinguish bona fide molecular dimers corresponding to crystallographic dimeric structures from looser molecular associations because of the uncertainty in localization. Therefore, we envision the following two possible explanations: 1) ErbB2 may slow down ErbB3 diffusion because of rearrangement of the submembrane actin meshwork or by inducing membrane deformation or folding (40). Although our TIR excitation strategy restricts the detection depth to ∼150 nm, less deep membrane undulations may lead to artifactual co-localizations and reduction of the apparent lateral diffusion coefficient (41). 2) ErbB2 co-expression may slow down ErbB3 homodimers by direct interactions (i.e., the formation of trimers or larger complexes). The fact that PRT treatment reduced the effect of ErbB2 co-expression on the homodimer fraction of ErbB3 and on ErbB3 mobility supports the hypothesis that direct involvement of ErbB2 in facilitating ErbB3 homodimerization may be partially behind these observations. The high fraction of ErbB3 in homo- and heterodimers, as determined in the two independent experimental settings, can also only be explained by the formation of complexes of ErbB3 homodimers and ErbB3-ErbB2 heterodimers. There is agreement in the literature that membrane curvature, retarded diffusion, and protein clustering in and beneath the membrane are causally related to each other in a self-organized system (35, 42). Although our results cannot pinpoint which of these effects is primarily caused by ErbB2 expression, retarded diffusion and enhanced formation of ErbB3 homodimers with or without membrane folding is obviously caused by ErbB2.

Bidirectional interactions between ErbB2 and the cytoskeleton were directly tested in our experiments. The diffusion of ErbB2 monomers is retarded by intact actin fibers because the disruption of microfilaments equalized the mobility of ErbB2 and ErbB3 by accelerating ErbB2 diffusion without having a significant effect on ErbB3. In addition, ErbB2 exerts a reciprocal effect on the very filaments retarding its diffusion by inducing the formation long actin filaments resembling stress fibers. Although such an effect of ErbB2 expression has not been reported to the best of our knowledge, ErbB2 is known to be involved in the formation of structures in which actin plays a role. It has been shown to be enriched in membrane protrusions (43), to induce tumor cell migration through Memo and cofilin (44), and to form linear chains in the plasma membrane due to presumed interactions with actin (45). The fact that ErbB2 overexpression induces membrane curvature (40), together with the interdependence of membrane shape, membrane protein clustering, and actin polymerization (35), sets the stage for the observed effect of ErbB2 on actin stress fibers and for the actin-dependent retardation of ErbB2 diffusion.

Besides membrane folding and cytoskeletal interactions, the expression level of ErbB3 and its interaction partners is also expected to influence clustering and the mobility of ErbB3. High expression level leads to high surface density, which favors the formation of clusters according to the law of mass action. It was the reason why the reported experiments were carried out with cells not expressing millions of ErbB3 on their surface, a circumstance that would have led to artifactually high dimer fractions. We attempted to correlate the surface density of ErbB3 with its dimer and immobile fraction as well as with its diffusion coefficient. Although there was an obvious tendency for the dimer fraction to be larger at high surface densities (data not shown), the curves were not quantitatively reproducible due to poor statistics (low fraction of dimers) and a multitude of other factors, which could also result in cell-to-cell variation in the dimer fraction and the diffusion coefficient of dimers. Therefore, we abandoned the cell-by-cell analysis, and the reported results for dimerization, immobile fractions, and diffusion coefficients were derived from pooled data sets, increasing the reliability of the measurements.

Our study also revealed that ErbB3 homodimers differ from ErbB3/ErbB2 heterodimers in two respects: 1) ErbB3/ErbB2 heterodimers were completely absent from PRT-pretreated samples, arguing that the dimerization arm of ErbB2 is indispensable for such heterocomplexes, whereas ErbB3 homodimerization was only partially inhibited by the anti-ErbB2 antibody, PRT. This latter finding confirms that ErbB2 facilitates the formation of ErbB3 homodimers and also implies that transient ErbB3/ErbB2 interactions might be involved in this effect (17). 2) Constitutive ErbB3 homodimers diffuse significantly faster than such ErbB3/ErbB2 heterodimers. Although the mobility of heterodimers is not significantly influenced by HRG treatment, the growth factor elicits a fourfold reduction in the diffusion coefficient of ErbB3 homodimers, rendering it similar to that of ErbB3/ErbB2 heterodimers. According to these results, three different classes of dimers could be distinguished: 1) low-mobility dimers (*D* ∼ 0.01–0.015 *μ*m^2^/s), including HRG-induced ErbB3 homodimers in the presence and absence of ErbB2 co-expression, and both constitutive and HRG-induced ErbB3/ErbB2 heterodimers; 2) intermediate-mobility dimers (D ∼ 0.03 *μ*m^2^/s), corresponding to constitutive ErbB3 homodimers in the presence of ErbB2 co-expression; and 3) high-mobility dimers (*D* ∼ 0.05 *μ*m^2^/s), corresponding to constitutive ErbB3 homodimers. Existence of these dimer classes is supported by statistical analysis (Table S4). We propose that cytoskeletal anchoring and the presence or absence of ErbB2-induced membrane folding, as explained in the previous paragraph, may be behind these differences. Alternatively, different kinds of forces or processes may be responsible for the formation of these dimer classes. Dimers assembled by direct molecular interactions and co-confined dimers (17, 38), held together by lipid-mediated or cytoskeletal interactions, are also expected to differ in terms of their mobility. Because our method does not allow us to differentiate between these dimeric species, the low- and high-mobility classes may also differ regarding the forces responsible for their assembly.

## Conclusions

**Figure 6:**
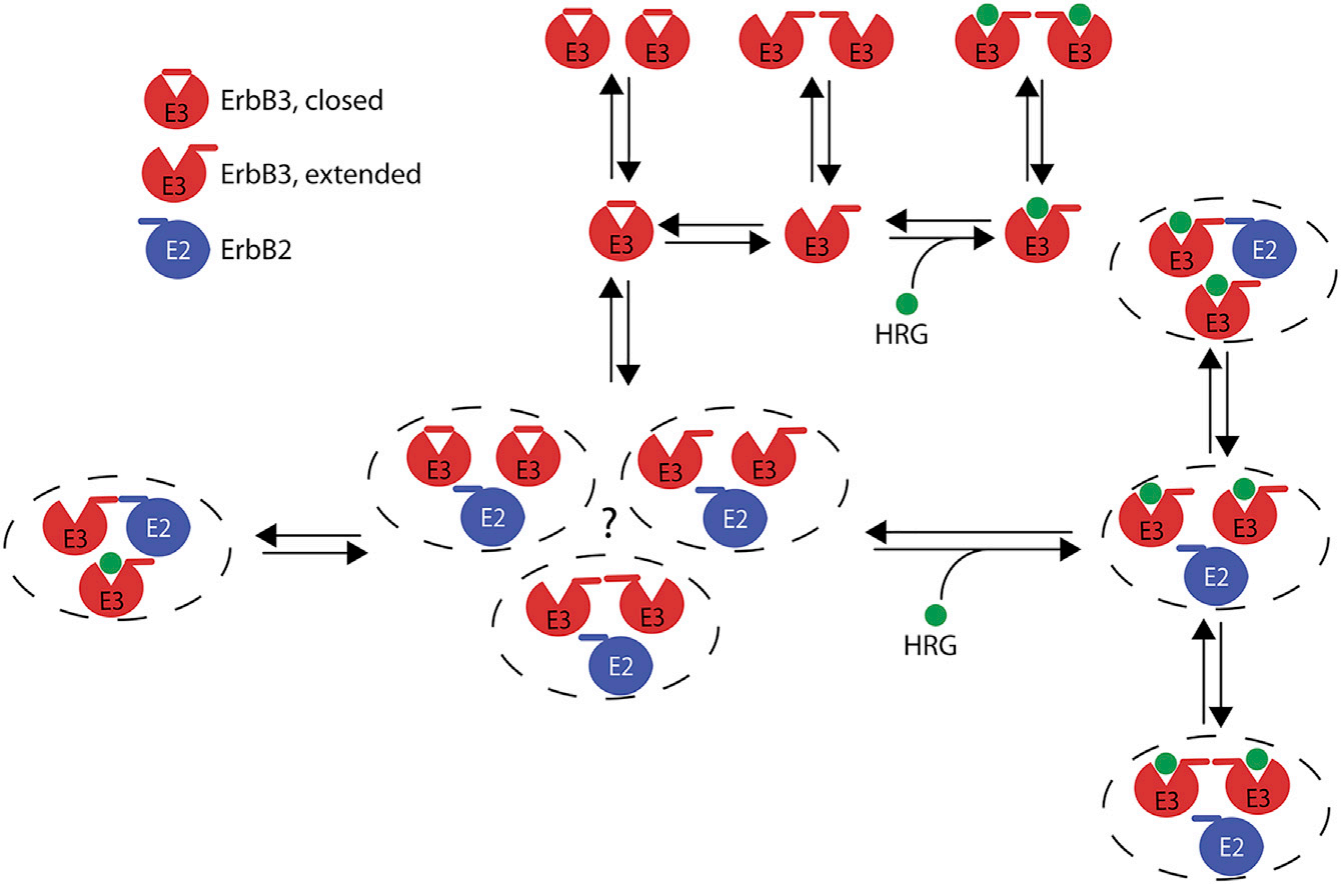
Model for the homo- and heteroclustering of ErbB3. The model encompasses all the evidence from tracking and co-localization measurements. ErbB3 is assumed to have a closed conformation in which the ligand binding site is blocked, and the dimerization arm is not exposed. The closed conformation is in equilibrium with the extended conformation, in which the ligand binding site is open, and the dimerization arm is exposed. Both liganded and unliganded ErbB3 in the extended conformation are assumed to undergo homodimerization, leading to the formation of bone fide homodimers. Besides these structures, looser ErbB3 homodimers may also form. ErbB2 competes with the formation of these bona fide ErbB3 homodimers, but it also facilitates the generation of ErbB3 homodimers (protein *symbols* surrounded by a *dashed ellipse*). The structure of ErbB2-induced ErbB3 homodimers is unclear. They can be bona fide ErbB3 homodimers or looser molecular associations. Formation of ErbB3/ErbB2 heterodimers is mediated by the dimerization arm of ErbB2 both in quiescent and HRG-stimulated cells because they are completely absent from PRT-pretreated samples. To see this figure in color, go online.

A model is proposed in which ErbB3 is assumed to be present in several different states (Fig. 6). The inactive form corresponds to the closed conformation of the extracellular domain. The extracellular domain of ErbB3 undergoes a conformational change to the active, extended state. Constitutive homodimers can be formed from the extended and closed state as well. These dimeric species are indistinguishable from each other by TOCCSL. It has already been suggested based on single particle tracking that besides dimers corresponding to crystallographic dimeric structures (10), looser, less stable dimers are also present (17, 38). The extended conformation of ErbB3 not only binds the ligand, HRG, but it also readily undergoes dimerization because of the exposure of the dimerization arm. Although not explicitly considered, mixed homodimers formed by a liganded and an unliganded ErbB3 could also be present, as suggested for ErbB1 previously (38). ErbB2 exerts a dual effect on ErbB3 homodimerization: 1) it retards the diffusion of ErbB3 homodimers and facilitates the formation of ErbB3 homodimers. The conformational state of ErbB3 in these dimers is uncertain because they can be bona fide homodimers or looser molecular associations. 2) ErbB2 also competes with the formation of bona fide ErbB3 homodimers stabilized by the dimerization arm. This effect is brought about by the formation of ErbB3/ErbB2 heterodimers stabilized by the dimerization arm of ErbB2. Such heterodimers are present in quiescent cells and in larger amounts in HRG-stimulated cells. ErbB2 is also involved in reciprocal interactions with the cytoskeleton because it induces the formation of long actin stress fibers, and its retarded diffusion, compared to ErbB3, is owed to intact actin filaments. The presented results offer a valuable insight into the workings of the ErbB3/ErbB2 receptor pair in a minimally perturbed system.

## Author Contributions

T.V. performed transfections and labeling of cells with antibodies and carried out and analyzed TOCCSL experiments. M.S. proposed and developed global analysis of homo- and heterodimerizations. E.S. supervised experiments and contributed ideas. D.K. proposed and developed the correction method for undersampling of slowly diffusing species. T.K., F.B., and G.B. generated plasmids and stably transfected cell lines. R.P. and J.B.H. performed and supervised flow cytometric experiments. J.S. advised about biological interpretation of results. G.J.S. interpreted experimental data and contributed important ideas. M.B. supervised the project and analyzed the data. P.N. conceived and supervised the project. T.V., G.J.S., M.B., and P.N. wrote the manuscript.

## Supporting Material

Document S1. Supporting Materials and Methods, Figs. S1–S4, and Tables S1–S4

Document S2. Article plus Supporting Material
