# Supplementary material for: Homo- and Heteroassociations Drive Activation of ErbB3
Source: Biophys J. 2019 Oct 9;117(10):1935–47. doi: 10.1016/j.bpj.2019.10.001 (PMC7018998; doi:10.1016/j.bpj.2019.10.001)
Supplement: Document S1. Supporting Materials and Methods, Figs. S1–S4, and Tables S1–S4 [file mmc1.pdf]

Biophysical Journal, Volume *117*

Supplemental Information

Homo- and Heteroassociations Drive Activation of ErbB3

Tímea Váradi, Magdalena Schneider, Eva Sevcsik, Dominik Kiesenhofer, Florian Baumgart, Gyula Batta, Tamás Kovács, René Platzer, Johannes B. Huppa, János Szöllősi, Gerhard J. Schütz, Mario Brameshuber, and Peter Nagy

Supplementary Methods

Determination of ErbB3-ErbB3 homodimer fractions in CHO-ErbB3 cell line: Binding of unlabeled ErbB3-Fab as well as binding of different amounts of AF488-ErbB3 Fab and AF647-ErbB3 Fab to ErbB3 yields an underestimation of determined ErbB3 homodimer fractions. By assuming the presence of plasma membrane structures containing either one or two ErbB3 molecule(s) and that all ErbB3 molecules are labeled by a Fab, the true number of homodimers can be estimated by a simple mathematical correction of experimentally determined apparent homodimer fractions. Observables in two-color TOCCSL experiments are the number of ErbB3 signals in the red and green color channel, S_R and S_G, the amount of visible ErbB3 homo-dimers corrected for false-positives, S_{coloc}, and the fraction of unlabeled ("white") Fabs, p_w.

For simultaneous labeling of ErbB3 via Fabs carrying red and green dyes at the same time, the overall probability p_w of a protein remaining unlabeled is estimated by

$$p_w = w_r \frac{S_R}{S_R+S_G} + w_g \frac{S_G}{S_R+S_G} \ , \qquad (1)$$

where w_r and w_g is the fraction of unlabeled ("white") protein in a sample labeled with either red or green dyes only (see section *Determination of the fraction of labeled Fabs bound to ErbB3*).

The probability of a protein being labeled red or green, respectively, is estimated by

$$p_r = (1-p_w)\frac{S_R}{S_R+S_G} \qquad \text{and} \qquad p_g = (1-p_w)\frac{S_G}{S_R+S_G} \, . \qquad (2)$$

Let *R*, *G* and *W* denote a red, green and white label, respectively. All possible combinations for labeling a dimer and their respective probabilities are given as follows:

Label	*RR*	*RG*	*RW*	*GR*	*GG*	*GW*	*WR*	*WG*	*WW*
Probability	p_r^2	$p_r p_g$	$p_r p_w$	$p_g p_r$	p_g^2	$p_g p_w$	$p_w p_r$	$p_w p_g$	p_w^2

In the two-color TOCCSL experiment only the labels *RG* and *GR* can be detected as co-localized spots, the number of which is given by S_{coloc}. All other dimer combinations are missed. Dimers carrying the labels *RR*, *RW* and *WR* are detected as red monomers, dimers labeled *GG*, *GW* or *WG* are detected as green monomers. *WW* dimers are not detected at all. Let D_{RR}, D_{RG}, D_{RW}, D_{GR}, D_{GG}, D_{GW}, D_{WR}, D_{WG} and D_{WW} denote the number of dimers in the sample that are labeled *RR*, *RG*, *RW*, *GR*, *GG*, *GW*, *WR*, *WG* and *WW*, respectively. The number of dimers in the sample is given by

$$D = \frac{S_{coloc}}{2p_r p_g} . \quad (3)$$

The numbers of red and green monomers, M_R and M_G, can be calculated as

$$M_R = S_R - S_{coloc} - D_{RR} - D_{RW} - D_{WR} \quad (4)$$

$$M_G = S_G - S_{coloc} - D_{GG} - D_{GW} - D_{WG} . \quad (5)$$

M_R and M_G represent the numbers of red and green labeled monomers, respectively, which can be written as

$$M_R = M \cdot p_r \quad \text{and} \quad M_G = M \cdot p_g \ , \quad (6)$$

where M is the total number of monomers in the sample. These equations are equivalent to

$$M = \frac{M_R}{p_r} = \frac{M_G}{p_g} . \quad (7)$$

In a real experiment, however, the red and green monomers might not be present in exactly this ratio. Therefore, the total number of monomers can be estimated by averaging the two values:

$$M = \frac{1}{2}\left(\frac{M_R}{p_r} + \frac{M_G}{p_g}\right) . \quad (8)$$

Finally, the relative frequency of dimers, i.e. the ErbB3-ErbB3 homodimer fraction in CHO-ErbB3 cells, is given by

$$D_{frac} = \frac{D}{M+D} . \quad (9)$$

Determination of ErbB3-ErbB3 homodimer fractions in CHO-ErbB2-ErbB3 cell line: The labeling procedure was the same as for CHO-ErbB3 cells, i.e. ErbB3 was competitively labeled with Fab fragments conjugated with either red or green dyes (AF647 and AF488).

Hence, the probability p_1^w of a protein remaining unlabeled is estimated in the same way as for the case of CHO-ErbB3 cells (see equation (1)). The probability p_1^r, p_1^g of an ErbB3 protein being labeled red or green, respectively, is estimated from the numbers of red and green signals, analogously to equation (2).

The amount of red and green signals S_1^R and S_1^G, respectively, is given by

$$S_1^R = M_R^{ErbB3} + D_{RR}^{hom} + D_{RG}^{hom} + D_{RW}^{hom} + D_{GR}^{hom} + D_{WR}^{hom} + D_{RW}^{het} \quad (10)$$

$$S_1^G = M_G^{ErbB3} + D_{RG}^{hom} + D_{GR}^{hom} + D_{GG}^{hom} + D_{GW}^{hom} + D_{WG}^{hom} + D_{GW}^{het} \ , \quad (11)$$

where M_R^{ErbB3}, M_G^{ErbB3} is the number of ErbB3 monomers labeled red or green, respectively, D_{RR}^{hom}, D_{GG}^{hom}, D_{RG}^{hom}, D_{GR}^{hom}, D_{RW}^{hom}, D_{WR}^{hom}, D_{GW}^{hom} and D_{WG}^{hom} are the numbers of ErbB3-ErbB3 homodimers carrying the labels denoted by the respective subscripts, and D_{RW}^{het}, D_{GW}^{het} are the numbers of ErbB3-ErbB2 heterodimers carrying the respective labels.

The number of colocalizations is given by

$$S_1^{coloc} = D_{RG}^{hom} + D_{GR}^{hom} = 2p_1^r p_1^g D^{hom} \ , \quad (12)$$

where D^{hom} is the total number of ErbB3-ErbB3 homodimers in the sample. This number can be obtained by rewriting equation (12) as

$$D^{hom} = \frac{S_1^{coloc}}{2p_1^r p_1^g} \ . \quad (13)$$

The number of ErbB3 monomers M^{ErbB3} and ErbB3-ErbB2 heterodimers D^{het} cannot be determined, because both are seen as just a red or just a green signal. We will obtain those numbers later by combining the data from the ErbB3-ErbB3 homodimer experiment with the data from the ErbB3-ErbB2 heterodimer experiment.

Determination of ErbB3-ErbB2 heterodimer fractions in CHO-ErbB2-ErbB3 cell line: For the ErbB3-ErbB2 heterodimer experiment we labeled the ErbB2 protein with a Fab fragment carrying a green dye (AF488) and the ErbB3 protein with a Fab fragment carrying a red dye (AF647).

We denote with $p_2^r \coloneqq \eta_r$ and $p_2^g \coloneqq \eta_g$ the probabilities of an ErbB3 protein being labeled red and an ErbB2 protein being labeled green, where η_r and η_g denote the corrected degree of labeling of ErbB3 and ErbB2, respectively (see section *Determination of the fraction of labeled Fabs bound to ErbB3*). The probability of an ErbB3 protein and ErbB2 protein remaining unlabeled ("white") is given by $p_2^{w_r} \coloneqq w_r$ and $p_2^{w_g} \coloneqq w_g$, where w_r and w_g denote the fraction of ErbB3 and ErbB2 proteins remaining without a dye ("white").

The amount of red signals S_2^R is given by

$$S_2^R = M_R^{ErbB3} + D_{RR}^{hom} + D_{RW}^{hom} + D_{WR}^{hom} + D_{RG}^{het} + D_{RW}^{het} \ , \quad (14)$$

where D_{RG}^{het} and D_{RW}^{het} are the numbers of ErbB3-ErbB2 heterodimers, where the ErbB3 protein carries a red label and the ErbB2 protein carries either a green or no ("white") label, respectively.

The number of colocalizations is given by

$$S_2^{coloc} = D_{RG}^{het} = p_2^r p_2^g D^{het}, \quad (15)$$

where D^{het} is the total number of ErbB3-ErbB2 heterodimers in the sample.

The number of heterodimers ErbB3-ErbB2 in the sample can thus be estimated by

$$D^{het} = \frac{S_2^{coloc}}{p_2^r p_2^g} \ . \quad (16)$$

The number of ErbB3 monomers M^{ErbB3} and ErbB3-ErbB3 homodimers D^{hom} cannot be determined, because both are seen just as red signals. We will obtain those numbers in the following by combining the data from the ErbB3-ErbB2 heterodimer experiment with the data from the ErbB3-ErbB3 homodimer experiment.

Combination of the ErbB3-ErbB3 homodimer and ErbB3-ErbB2 heterodimer experiments: We assume that the proportions of ErbB3 monomers, ErbB3-ErbB3 homodimers and ErbB3-ErbB2 heterodimers are the same in both experiments. Hence, it holds that

$$M_1^{ErbB3}/D_1^{hom} = M_2^{ErbB3}/D_2^{hom} \tag{17}$$

$$M_1^{ErbB3}/D_1^{het} = M_2^{ErbB3}/D_2^{het} \;, \tag{18}$$

where the subscripts 1 and 2 indicate the numbers from the homodimer and heterodimer experiments, respectively. From the experiments, we know the number of red signals S_1^R, S_2^R, green signals S_1^G, S_2^G and the number of colocalized spots S_1^{coloc}, S_2^{coloc}. We want to determine the amounts of monomers M_1^{ErbB3}, M_2^{ErbB3}, ErbB3-ErbB3 homodimers D_1^{hom}, D_2^{hom} and ErbB3-ErbB2 heterodimers D_1^{het}, D_2^{het} in both experiments.

From equations (13) and (16) we know that

$$D_1^{hom} = \frac{S_1^{coloc}}{2p_1^r p_1^g} \qquad \text{and} \qquad D_2^{het} = \frac{S_2^{coloc}}{p_2^r p_2^g} \;. \tag{19}$$

Taking together equations (10) and (11) from the homodimer experiment we obtain

$$S_1^R + S_1^G - D_1^{hom}\left(p_1^r p_1^r + 2p_1^r p_1^w + 4p_1^r p_1^g + 2p_1^g p_1^w + p_1^g p_1^g\right) = M_1^{ErbB3}(p_1^r + p_1^g) + D_1^{het}(p_1^r + p_1^g) \;. \tag{20}$$

Rearranging equation (14) from the heterodimer experiment leads to

$$S_2^R - D_2^{het}\left(p_2^r p_2^g + p_2^r p_2^{w_g}\right) = M_2^{ErbB3} p_2^r + D_2^{hom}(p_2^r p_2^r + 2p_2^r p_2^{w_r}) \;. \tag{21}$$

Assuming that all the variables are nonzero, we can solve this system of equations (17)-(21) to obtain the unknown variables M_1^{ErbB3}, M_2^{ErbB3}, D_1^{hom}, D_2^{hom}, D_1^{het} and D_2^{het}.

Finally, the fractions of ErbB3 proteins present in monomers or bound either in homodimers or heterodimers can be given as follows:

$$M_{frac}^{ErbB3} = \frac{M^{ErbB3}}{M^{ErbB3} + 2D^{hom} + D^{het}} \tag{22}$$

$$D_{frac}^{hom} = \frac{2D^{hom}}{M^{ErbB3} + 2D^{hom} + D^{het}} \tag{23}$$

$$D_{frac}^{het} = \frac{D^{het}}{M^{ErbB3} + 2D^{hom} + D^{het}} \;, \tag{24}$$

where the fractions are the same inserting either results for M^{ErbB3}, D^{hom}, D^{het} from the homodimer experiment (subscript 1) or heterodimer experiment (subscript 2).

Determination of the fraction of labeled Fabs bound to ErbB3: Assuming a Poissonian labeling distribution of the number of dyes per Fab fragment and that only Fabs with 1 or 2 dyes are able to bind to their target [(1)], the fractions of unlabeled, x_0, and labeled, x_{dye}, Fab fragments are given by

$$x_0 = \frac{P(0,DOL)}{\sum_{i=0}^{2} P(i,DOL)} \tag{25}$$

$$x_{dye} = 1 - x_0. \tag{26}$$

Here, DOL is the spectroscopically determined degree of labeling and equals the average number of dyes per Fab fragment.

Let's assume the following binding scheme

$$ErbB3Fab_0 \underset{k_{0+}}{\overset{k_{0-}}{\rightleftarrows}} ErbB3 \underset{k_{dye+}}{\overset{k_{dye-}}{\leftrightarrows}} ErbB3Fab_{dye} \tag{27}$$

with $ErbB3Fab_0$ representing an unlabeled Fab bound to ErbB3, $ErbB3$ the protein without any Fab, $ErbB3Fab_{dye}$ an dye-labeled Fab bound to ErbB3 and k_{0-}, k_{0+}, k_{dye-} and k_{dye+} the respective rates. The differential equations for this binding scheme can be written as

$$\frac{d[ErbB3]}{dt} = k_{0-}[ErbB3Fab_0] + k_{dye-}[ErbB3Fab_{dye}] - k_{0+}[ErbB3].[Fab_0] - k_{dye+}[ErbB3].[Fab_{dye}] \tag{28}$$

$$\frac{d[ErbB3Fab_0]}{dt} = k_{0+}[ErbB3].[Fab_0] - k_{0-}[ErbB3Fab_0] \tag{29}$$

$$\frac{d[ErbB3Fab_{dye}]}{dt} = k_{dye+}[ErbB3].[Fab_{dye}] - k_{dye-}[ErbB3Fab_{dye}]. \tag{30}$$

In equilibrium, it holds that

$$\frac{d[ErbB3]}{dt} = \frac{d[ErbB3Fab_0]}{dt} = \frac{d[ErbB3Fab_{dye}]}{dt} = 0 \quad . \tag{31}$$

Hence, in equilibrium it follows from (29) that

$$\frac{k_{0+}}{k_{0-}} = \frac{[ErbB3Fab_0]}{[ErbB3].[Fab_0]} = \frac{1}{K_{D0}} \tag{32}$$

with K_{D0} being the equilibrium dissociation constant for an unlabeled Fab.

From (30) it follows that

$$\frac{k_{dye+}}{k_{dye-}} = \frac{[ErbB3Fab_{dye}]}{[ErbB3].[Fab_{dye}]} = \frac{1}{K_{Ddye}} \tag{33}$$

with K_{Ddye} being the equilibrium dissociation constant for a dye-conjugated Fab.

Dividing (32) by (33) yields

$$\frac{[ErbB3Fab_0]}{[ErbB3Fab_{dye}]} = \frac{K_{Ddye}}{K_{D0}} \frac{[ErbB3].[Fab_0]}{[ErbB3].[Fab_{dye}]} = \frac{K_{Ddye}}{K_{D0}} \frac{[Fab_0]}{[Fab_{dye}]} \quad . \tag{34}$$

Hence, the ratio of bound labeled and unlabeled Fabs yields

$$\frac{[ErbB3Fab_{dye}]}{[ErbB3Fab_0]} = \frac{K_{D0}}{K_{Ddye}} \frac{[Fab_{dye}]}{[Fab_0]} = \frac{K_{D0}}{K_{Ddye}} \frac{x_{dye}}{x_0}. \tag{35}$$

Finally, the fraction of ErbB3 being labeled by a Fab carrying one or two dyes yields

$$\frac{[ErbB3Fab_{dye}]}{[ErbB3Fab_{dye}]+[ErbB3Fab_0]} = \frac{K_{D0}x_{dye}}{K_{D0}x_{dye}+K_{Ddye}x_0} := \eta. \tag{36}$$

η can be interpreted as the corrected degree of labeling of ErbB3 accounting for unlabeled Fab fragments and was further used for correcting apparent dimer values. The same calculation is valid for Fab fragments used for labeling ErbB2 in the heterodimer experiment. In either case, η_r (η_g) refers to the probability of labeling a protein with an Alexa647 (Alexa488) -conjugated Fab fragment (i.e. the apparent labeling efficiency). The fraction w of proteins in a sample remaining without a dye ("white" proteins) is given by $w = 1 - \eta$. Numbers for equilibrium constants, DOL and the probabilities can be found in Supplemental Table 1.

Correction of ErbB3 homodimer fractions due to different diffusion constants: Assuming a difference in mobility of ErbB3 monomers and dimers (diffusion coefficients D_1 and D_2), the apparent result of a TOCCSL experiment underestimates the fraction with lower mobility – in our case the fraction of ErbB3 homodimers (see **Table 1** for values of diffusion constants). After the recovery phase, dimers with decreased mobility have a lower probability to be detected in the analysis region compared to faster monomers. Based on known diffusion coefficients, the results can be reevaluated and a corrected value for the homoassociation can be determined.

The true dimer fraction, as presented in the results, is estimated by approximating the recovery process in the two-color-TOCCSL experiment by means of an analytical calculation for a slit aperture with an infinite extension in the y-direction. Consequently, variations occur only along the x-axis.

Assuming an aperture with infinitely sharp edges at position ±dx/2, an ideal photobleaching process and free Brownian motion of the labeled molecules, the surface density profile along the x-axis for a molecule species i with diffusion coefficient D_i after the recovery time t_{rec} is given by

$$\rho_i(x,t) = \rho_{0,i}\left[1 - \frac{1}{2}\mathrm{erf}\left(\frac{x+dx/2}{\sqrt{4D_i t}}\right) + \frac{1}{2}\mathrm{erf}\left(\frac{x-dx/2}{\sqrt{4D_i t}}\right)\right] \tag{37}$$

where erf denotes the Gaussian error function and $\rho_{0,i}$ the initial surface density in the shielded part of the membrane. The initial overall surface density ρ_{fl} (unit: fluorophores/µm²) including both color channels is composed of the initial monomer and dimer surface densities $\rho_{fl} = \rho_{0,monomer} + 2 \cdot \rho_{0,dimer}$. The surface densities $\rho_{0,monomer}$ and $\rho_{0,dimer}$ (unit: oligomers/µm²) can easily be calculated from ρ_{fl} for a given stoichiometric composition of monomers and dimers. Eq. 37 is then used to calculate the monomer and dimer surface density profiles $\rho_{monomer}(x,t_{rec})$ and $\rho_{dimer}(x,t_{rec})$ for the initial densities $\rho_{0,monomer}$ and $\rho_{0,dimer}$ respectively, after a given recovery time t_{rec}.

The apparent dimer fraction is determined as the ratio of the number of dimers N_{dimer} and the total number of molecules N_{total} in the analysis region

$$F_{dimer}^{app} = \frac{N_{dimer}}{N_{total}} = \frac{\int_0^{x_{cutoff}} \rho_{dimer}(x,t=t_{rec})\, dx}{\int_0^{x_{cutoff}} \rho_{total}(x,t=t_{rec})\, dx}. \qquad (38)$$

The analysis region is confined in x-direction by the limit $\pm x_{cutoff}$, where the surface density exceeds a given cutoff surface density $\rho_{cutoff} = \rho_{channel,j}(x_{cutoff}, t_{rec})$. The index j=1 or 2 denotes the color channel with the higher surface density, assuming non-equimolar labeling conditions. Here, a value of ρ_{cutoff} = 1 signal/µm², representing the single molecule detection limit, is used for the calculations. Thus, in order to determine x_{cutoff}, the surface density profile in oligomers/µm² in the channel with the higher surface density is calculated as

$$\rho_{channel,j}(x,t) = \rho_{monomer,j}(x,t) + \rho_{dimer1,j}(x,t) + \rho_{dimer12,j}(x,t) \qquad (39)$$

with $\rho_{monomer}$ the monomer density, $\rho_{dimer1,j}$ the density of dimers present in only one color channel (i.e. a dimer labeled with the same color), and $\rho_{dimer12,j}$ the density of dimers visible in both color channels, $\rho_{monomer,j}$ (i.e. a dimer labeled with both colors).

Eq. 39 leads to

$$\rho_{channel,1or2}(x,t) = \rho_{monomer}(x,t)\, p_{channel,1or2} + \rho_{dimer}(x,t)\left[p_{channel,1or2}^2 + 2\, p_{channel,1} \cdot p_{channel,2}\right] \qquad (40)$$

with $p_{channel,j}$ representing the labeling probabilities for two spectrally distinct fluorescent labels.

The apparent dimer fraction $F_{app} = F_{app}(f_{true})$ in an ideal two-color TOCCSL experiment is thereby determined as a function of the true dimer fraction f_{true} (Supplemental Figure 4A). The calculations show that the apparent dimer fraction approaches the real

dimer fraction as $t_{rec}\rightarrow\infty$ (Supplemental Figure 4B). By evaluating the interpolated and inverted calculated apparent dimer fraction F_{app}^{-1} at the value of the experimentally obtained apparent dimer fraction $F_{experiment}$, the true dimer fraction in the TOCCSL experiment f_{true}^{exp} can be obtained:

$$f_{true}^{exp} = F_{app}^{-1}\left(F_{experiment}\right). \tag{41}$$

For every TOCCSL experiment, $F_{app}(f_{true})$ was calculated based on the experimentally determined parameters D_1, D_2 and ρ_0, and f_{true}^{exp} was determined.

Supplemental figures and tables

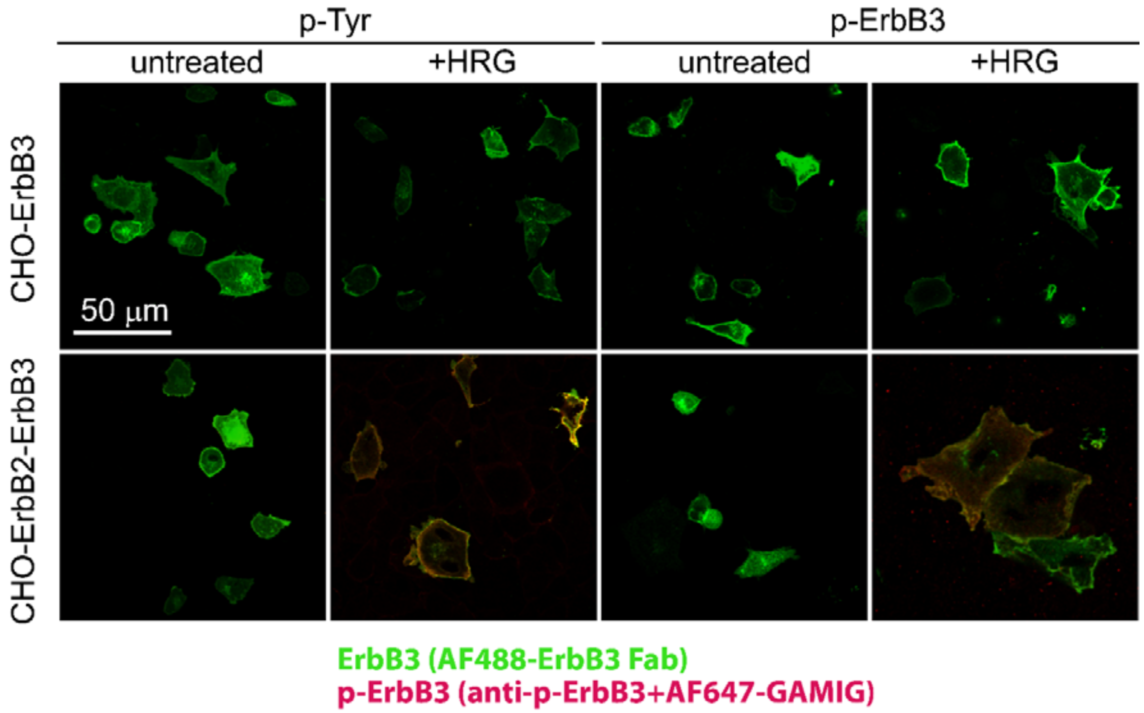

Supplemental Figure 1. Measurement of constitutive and HRG-induced tyrosine phosphorylation in the cell lines used for two-color TOCCSL experiments

CHO and CHO-ErbB2 cells were transiently transfected with ErbB3 generating the CHO-ErbB3 and CHO-ErbB2-ErbB3 cell lines, respectively. Cells were serum-starved overnight followed by treatment with 25 nM heregulin (HRG) at 37°C. Control and stimulated cells were fixed, permeabilized and labeled with primary monoclonal antibodies against phospho-tyrosine (p-Tyr) or ErbB3 phosphorylated at Tyr1289 (p-ErbB3) followed by staining with AF647-conjugated secondary antibodies (GAMIG – goat anti-mouse, red channel of images). The green channel corresponds to the signal of the AF488-anti-ErbB3 Fab. Images of both color channels were recorded separately using a confocal microscope and overlaid for display.

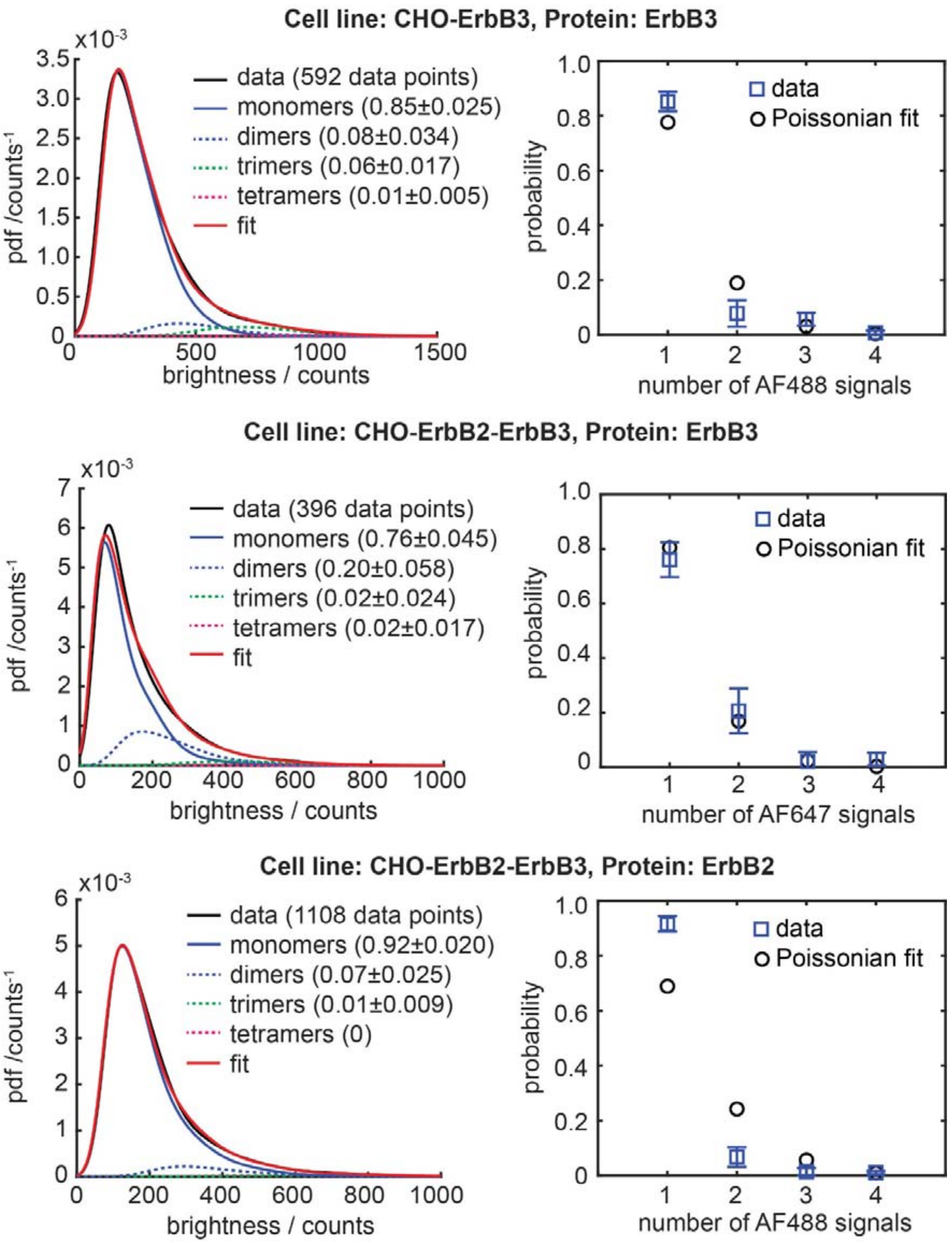

Supplemental Figure 2. Brightness analysis of ErbB2 and ErbB3 detected in an individual color channel shows no indication of larger clusters.

The brightness distribution of single fluorescence spots in CHO-ErbB3 and CHO-ErbB2-ErbB3 cells after HRG stimulation is plotted as a probability density function (left). Data (black line)

were fitted as described in ref. (2) (red line) with the single-molecule brightness reference taken from the last tracking image. The fits yielded an overwhelming majority of monomeric species for all experimental conditions. The percent values of monomeric, dimeric, trimeric and tetrameric species, determined from these fits, are shown as blue squares in the plots on the right. Subsets of all data were randomly selected 100-times, fitted and the plotted values represent the means and standard errors of these fittings. For comparison, the number of dyes per Fab considering a Poissonian distribution was calculated with the “true degree of labeling” (see Supplemental Table 1, black circles in the plot on the right). These results exclude the presence of a significant fraction of higher-order oligomeric ErbB3 species in the TOCCSL images.

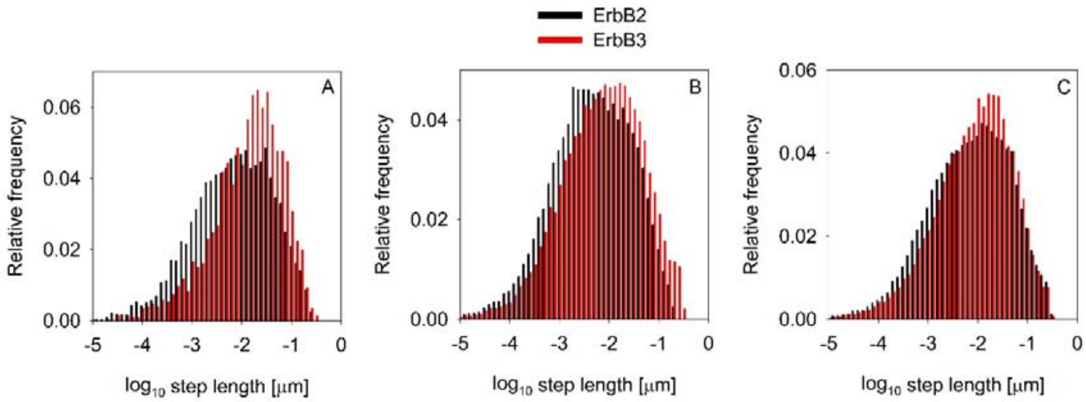

Supplemental Figure 3. Distribution of step lengths in single particle tracking

Single particle tracking of fluorescent Fab-labeled cells was carried out, and the distribution of step lengths under different experimental conditions is shown as normalized histograms.

A. Step length distribution of ErbB2 and ErbB3 in untreated CHO-ErbB2-ErbB3 cells generated from data displayed in Table 1 of the manuscript.

B-C. In a different experiment control CHO-ErbB2-ErbB3 cells (B) and those treated with latrunculin B (C) were analyzed.

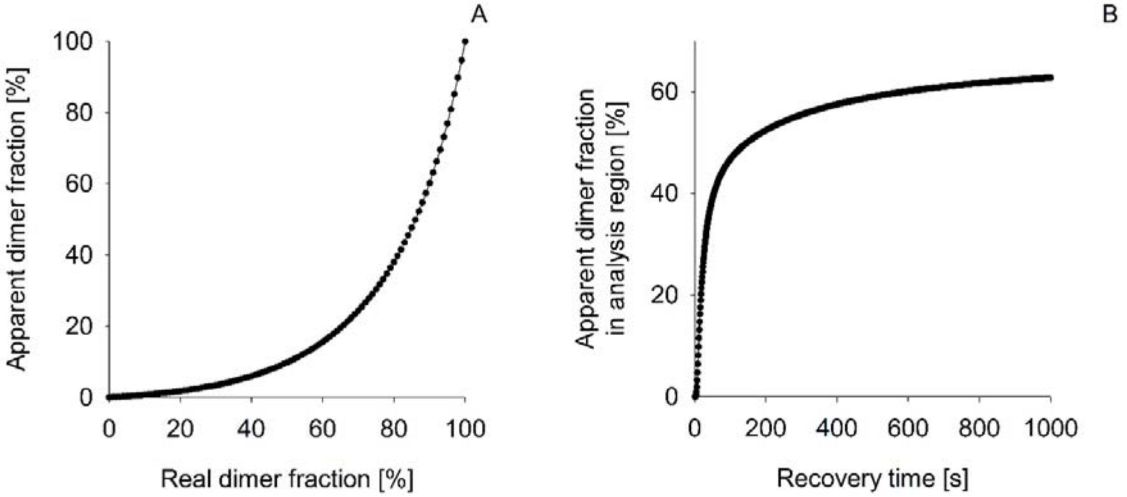

Supplemental Figure 4. Alterations of apparent ErbB3 homodimer fractions due to different diffusion constants of monomeric and dimeric species

A. TOCCSL experiments were simulated assuming different real dimer fractions according to equations 37-41. The apparent dimer fractions were calculated according to equation 38 considering different diffusion constants for monomers and dimers. The following parameters were used for the calculations shown: aperture size 10 µm; diffusion constants for monomers and dimers D_1 = 0.1 µm²/s and D_2 = 0.05 µm²/s, respectively; density of ErbB3 molecules ρ_0 = 100 molecules/µm²; recovery time in the TOCCSL experiment t_{rec} = 5s.

B. Change in the apparent dimer fraction as a function of recovery time. A TOCCSL experiment was simulated assuming a 70% real dimer fraction and diffusion constants D_1 = 0.05 µm²/s and D_2 = 0.02 µm²/s for moners and dimers, respectively. The apparent dimer fraction, calculated as in part A, approaches the real dimer fraction for $t \rightarrow \infty$.

Supplemental Table 1. Characterization of probes used for fluorescent labeling

Probe	***DOL***	K_{D0} **(*µg/ml*)**	K_{D488} **(*µg/ml*)**	K_{D647} **(*µg/ml*)**	η_r	η_g
AF488-ErbB3-Fab	1.4	4	10	-	-	0.488
AF647-ErbB3-Fab	1.8	4	-	19	0.419	-
AF488-ErbB2-Fab	1.4	4.6	4.6	-	-	0.704

The degree of labeling (DOL) was characterized by absorption spectroscopy. The dissociation constants of unlabeled Fab (K_{D0}), AF488-labeled Fab (K_{D488}) and AF647-labeled Fab (K_{D647}) were determined by fitting of concentration dependent, equilibrium binding data, obtained by flow cytometry, as described in *Experimental procedures*. The probability that an ErbB2 protein is labeled by a red, AF647-tagged Fab (η_r) and the probability that ErbB3 is labeled by a green, AF488-conjugated Fab (η_g) were calculated as described in *Supplementary Methods*.

Supplemental Table 2. Percentages of homo- and heterodimers under different experimental conditions

			Raw data		Correction for labeling		Correction for diffusion		Joint analysis	
	HRG	PRT	ErbB3 homo	ErbB3/2 hetero	ErbB3 homo	ErbB3/2 hetero	ErbB3 homo	ErbB3/2 hetero	ErbB3 homo	ErbB3/2 hetero
CHO-ErbB3 cells	–	–	1.1% ±0.5		3% ±1.3%		13.2% ±4.8%			
	–	+								
	+	–	3.7% ±0.5		13% ±2.6%		69.5% ±3.9%			
	+	+								
CHO-ErbB2-ErbB3 cells	–	–	1.6% ±1%	2.4% ±0.4%	3.5% ±2%	4.4% ±0.6%	54% ±9%	70.8% ±1.6%	22% ±6%	34% ±7%
	–	+	3.6% ±0.5%	0.4% ±0.1%	8.7% ±1.3%	0.6% ±0.1%	15% ±2%	0.8% ±0.1%	14% ±1%	1% ±1%
	+	–	6.8% ±1.5%	4.4% ±0.5%	16.2% ±4%	8.1% ±0.9%	77.8% ±3.5%	88.4% ±0.6%	45% ±5%	43% ±11%
	+	+	5.1% ±0.3%	1.5% ±0.1%	13.2% ±2.2%	2.9% ±0.2%	36% ±3.9%	14.5% ±1%	27% ±2%	3% ±1%

Dimeric events were determined from colocalizations in two-color TOCCSL experiments, and these numbers were normalized to the total number of ErbB3 signals detected under the given experimental condition ("raw data"). These percentages were corrected for unlabeled ErbB2 or ErbB3 (i.e. ErbB3/2 bound to an Fab without a fluorophore), for the lower binding affinity of labeled Fabs compared to unlabeled ones and for different fractions of the two antibodies applied in all ErbB3 homo-association experiments ("correction for labeling"), followed by taking the slower diffusion of dimers into consideration ("correction for diffusion"). The results of homo- and heterodimerization experiments in CHO-ErbB2-ErbB3 cells were pooled to obtain the percentages of homo- and heterodimers as a function of ErbB3 ("joint analysis"). The numbers shown represent the mean±SEM.

Supplemental Table 3. Statistical analysis of the differences between dimer fractions

ErbB3 homoassociation (all dimers)			CHO-ErbB3		CHO-ErbB2-ErbB3		
			no HRG	+ HRG	no HRG<br>no PRT	+ HRG<br>no PRT	no HRG<br>+ PRT
CHO-ErbB3	no HRG						
	+ HRG		0.001<br>effect of HRG in the absence of ErbB2				
CHO-ErbB2-ErbB3	no HRG	no PRT	0.001<br>effect of ErbB2 co-expression	0.627<br>effect of ErbB2 co-expression on the HRG effect			
	+ HRG	no PRT	0.001	0.995	0.08<br>effect of HRG		
	no HRG	+ PRT	1.000	0.001	0.002<br>effect of PRT without HRG stimulation	0.001	
	+ HRG	+ PRT	0.11	0.003	0.395	0.001<br>effect of PRT on HRG stimulation	0.187

ErbB3 heteroassociation (all dimers)			CHO-ErbB2-3		
			no HRG<br>no PRT	+ HRG<br>no PRT	no HRG<br>+ PRT
CHO-ErbB2-ErbB3	no HRG	no PRT			
	+ HRG	no PRT	0.769<br>effect of HRG		
	no HRG	+ PRT	0.014<br>effect of PRT without HRG stimulation	0.004	
	+ HRG	+ PRT	0.021	0.004<br>effect of PRT on HRG stimulation	0.997

The *p* values in the body of the table, characterizing the statistical significance of the differences in dimer percentages shown in Fig. 2, were determined with Tukey's HSD test in order to control the familywise error rate.

Supplemental Table 4. Statistical analysis of the diffusion coefficients of dimers

	ErbB3/ErbB3, constitutive, no ErbB2 D=0.052 $\mu m^2/s$	ErbB3/ErbB3, constitutive, with ErbB2 D=0.031 $\mu m^2/s$	ErbB3/ErbB3, HRG-induced, no ErbB2 D=0.014 $\mu m^2/s$	ErbB3/ErbB3, HRG-induced, with ErbB2 D=0.018 $\mu m^2/s$	ErbB3/ErbB2, constitutive D=0.017 $\mu m^2/s$	ErbB3/ErbB2, HRG-induced D=0.01 $\mu m^2/sec$
ErbB3/ErbB3, constitutive, no ErbB2 D=0.052 $\mu m^2/s$		0.0003	0.0003	0.0003	0.0003	0.0003
ErbB3/ErbB3, constitutive, with ErbB2 D=0.031 $\mu m^2/s$			0.0003	0.0005	0.002	0.0007
ErbB3/ErbB3, HRG-induced, no ErbB2 D=0.014 $\mu m^2/s$				0.65	0.95	0.96
ErbB3/ErbB3, HRG-induced, with ErbB2 D=0.018 $\mu m^2/s$					0.99	0.46
ErbB3/ErbB2, constitutive D=0.017 $\mu m^2/s$						0.71
ErbB3/ErbB2, HRG-induced D=0.01 $\mu m^2/s$						

The *p* values in the body of the table, characterizing the statistical significance of the differences in the diffusion coefficients of dimers shown in Table 1, were determined with Tukey's HSD test in order to control the familywise error rate. Cells corresponding to statistically significant differences are shaded.

Supplementary references

1. Szabó, A., Szendi-Szatmári, T., Ujlaky-Nagy, L., Rádi, I., Vereb, G., Szöllősi, J., and Nagy, P. (2018) The effect of fluorophore conjugation on antibody affinity and the photophysical properties of dyes. *Biophys J* **114**, 688-700
2. Brameshuber, M., Weghuber, J., Ruprecht, V., Gombos, I., Horváth, I., Vigh, L., Eckerstorfer, P., Kiss, E., Stockinger, H., and Schütz, G. J. (2010) Imaging of mobile long-lived nanoplatforms in the live cell plasma membrane. *J Biol Chem* **285**, 41765-41771
